# Approaches of wearable and implantable biosensor towards of developing in precision medicine

**DOI:** 10.3389/fmed.2024.1390634

**Published:** 2024-07-18

**Authors:** Elham Ghazizadeh, Zahra Naseri, Hans-Peter Deigner, Hossein Rahimi, Zeynep Altintas

**Affiliations:** ^1^Department of Bioinspired Materials and Biosensor Technologies, Faculty of Engineering, Institute of Materials Science, Kiel University, Kiel, Germany; ^2^Department of Medical Biotechnology, School of Medicine, Mashhad University of Medical Sciences, Mashhad, Iran; ^3^Institute of Precision Medicine, Furtwangen University, Villingen-Schwenningen, Germany; ^4^Fraunhofer Institute IZI (Leipzig), Rostock, Germany; ^5^Faculty of Science, Eberhard-Karls-University Tuebingen, Tuebingen, Germany; ^6^Department of Medicine, University of Pittsburgh, Pittsburgh, PA, United States

**Keywords:** wearable, implantable, biosensors, precision medicine, health

## Abstract

In the relentless pursuit of precision medicine, the intersection of cutting-edge technology and healthcare has given rise to a transformative era. At the forefront of this revolution stands the burgeoning field of wearable and implantable biosensors, promising a paradigm shift in how we monitor, analyze, and tailor medical interventions. As these miniature marvels seamlessly integrate with the human body, they weave a tapestry of real-time health data, offering unprecedented insights into individual physiological landscapes. This log embarks on a journey into the realm of wearable and implantable biosensors, where the convergence of biology and technology heralds a new dawn in personalized healthcare. Here, we explore the intricate web of innovations, challenges, and the immense potential these bioelectronics sentinels hold in sculpting the future of precision medicine.

## Introduction

Precision medicine represents a paradigm shift in healthcare, aiming to tailor medical interventions to individual characteristics, allowing for more personalized and effective treatments. The role of precision medicine in developing healthcare and treatment is not just about individualized care; it signifies a paradigm shift towards a more proactive, informed, and patient-centric healthcare system (1). As our understanding of genetics deepens and technology continues to evolve, precision medicine will undoubtedly play an increasingly pivotal role in shaping the future of healthcare, offering a level of precision and effectiveness that was once deemed the stuff of futuristic visions. In this new era, healthcare is not just about treating diseases; it’s about understanding and optimizing the unique genetic tapestry of each individual for a healthier, more resilient tomorrow (2). Wearable and implantable biosensors have emerged as key enablers in this transformative journey, offering continuous, real-time monitoring of physiological parameters. On the other front, wearable biosensors have ushered in a new era of healthcare by providing a continuous stream of data about an individual’s physiological status. These devices, ranging from smartwatches and fitness trackers to more specialized medical-grade sensors, can monitor parameters such as heart rate, blood pressure, glucose levels, and even more complex metrics like ECG patterns. Wearable biosensors offer a real-time, comprehensive view of a person’s health, enabling early detection of anomalies and facilitating proactive healthcare interventions (3).

Wearable biosensors, integrated into clothing or accessories, provide a non-intrusive means of collecting a wealth of data, including vital signs, activity levels, and even biochemical markers. These devices empower individuals to actively participate in their healthcare, fostering a proactive approach to well-being. Moreover, the seamless integration of data from wearables into electronic health records facilitates a comprehensive understanding of a patient’s health status (4).

Implantable biosensors, on the other hand, delve deeper into the intricacies of the human body, offering unprecedented access to internal physiological processes. These miniaturized marvels are designed to monitor specific biomarkers, providing clinicians with intricate insights into disease progression and treatment response. The potential for early detection of anomalies and swift intervention holds promise for preventing diseases before they manifest clinically (5).

The synergy between wearable and implantable biosensors contributes to a holistic approach to precision medicine. Continuous data streams from wearables serve as a foundation for baseline health, while implantable offer focused, in-depth information on specific parameters. Integrating this wealth of data through advanced analytics and artificial intelligence not only refines diagnostics but also enhances predictive modeling for treatment outcomes (6). The amalgamation of real-time, personalized data from these devices holds the promise of transforming healthcare from reactive to proactive, offering a future where medical interventions are precisely tailored to individual needs, optimizing outcomes, and improving the overall quality of life. This review explores the pivotal role of these biosensors in advancing precision medicine.

## Precision medicine and wearable/implantable biosensor

In the relentless pursuit of improving healthcare outcomes, precision medicine has emerged as a revolutionary paradigm, challenging the conventional one-size-fits-all approach. Unlike traditional medicine, which often employs generalized treatments, precision medicine tailor’s healthcare strategies to the unique genetic, environmental, and lifestyle characteristics of each individual. This bespoke approach not only transforms the landscape of diagnosis and treatment but holds the potential to redefine the entire healthcare experience (7, 8). Armed with genetic insights, healthcare providers can identify potential health risks long before symptoms manifest. This early detection empowers a proactive approach to healthcare, where interventions can be implemented to prevent the onset of diseases or manage them at their earliest stages (9). This knowledge allows healthcare professionals to prescribe drugs that are not only effective but also tailored to each patient, minimizing the trial-and-error process often associated with medication regimens. Precision medicine contributes to a deeper understanding of diseases at the molecular level. This knowledge not only aids in more accurate diagnoses but also fuels ongoing research, leading to the development of innovative therapies that target the specific mechanisms driving diseases (10).

In the ever-evolving landscape of healthcare, the convergence of precision medicine and wearable biosensors has emerged as a groundbreaking frontier, promising personalized and real-time insights into an individual’s health. Precision medicine, which tailors medical care to the unique characteristics of each patient, and wearable biosensors, compact devices that continuously monitor physiological parameters, are joining forces to revolutionize how we approach diagnosis, treatment, and overall healthcare management (11).

**Figure 1:**
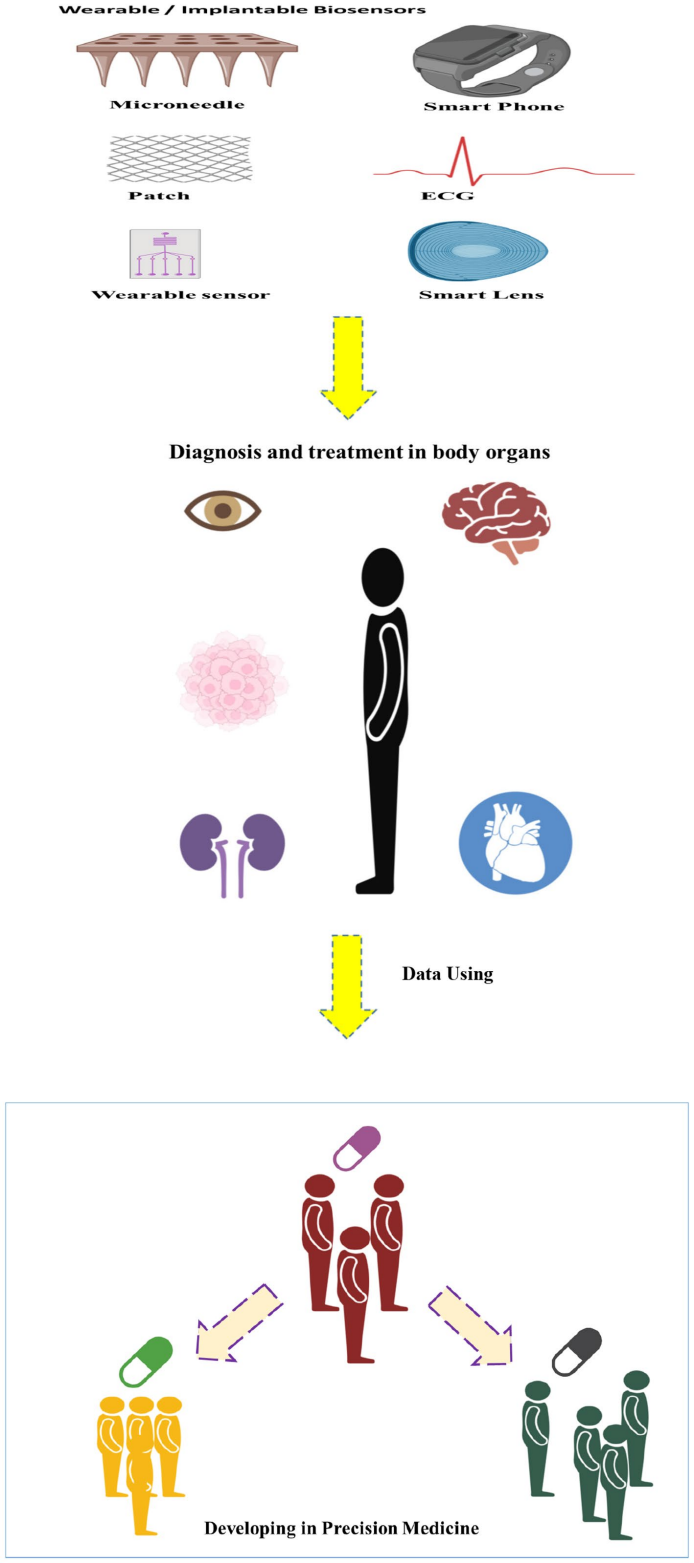
Schematic image of developing of the wearable/implantable biosensors in the precision medicine.

When precision medicine and wearable biosensors intersect, the synergy created is nothing short of transformative. Imagine a scenario where a patient’s genetic predisposition to a particular condition is combined with real-time data from wearable biosensors, allowing healthcare providers to predict, prevent, or manage diseases with unprecedented accuracy (12, 13). By integrating genetic data with continuous monitoring, healthcare professionals can identify early signs of diseases or health risks, enabling preventive measures to be implemented before symptoms manifest. Personalized treatment plans, informed by both genetic insights and real-time physiological data, ensure that interventions are tailored to an individual’s unique profile. This targeted approach enhances treatment efficacy and reduces the risk of adverse reactions (14, 15). Wearable biosensors enable remote patient monitoring, allowing healthcare providers to track a patient’s health in real-time without the need for frequent clinic visits (16). This is particularly beneficial for individuals with chronic conditions or those recovering from surgery. The wealth of data generated by wearable biosensors, when analyzed in conjunction with genetic information, contributes to a deeper understanding of the factors influencing health outcomes (17). This data-driven approach facilitates ongoing research, leading to continuous improvements in precision medicine (Figure 1). Here, we have compiled a summary of recent advances in the development of wearable biosensors for monitoring health and disease-related symptoms with the aim of ameliorating various health-threatening diseases in different organs of the body.

## Wearable/implantable technology and applications in cardiovascular care

In 2003, Cardionet Inc. developed the first mobile telemetry system, which recorded ECG and uploaded ambulatory data via cellular connections. It also contained algorithms for automatically transmitting and detecting asymptomatic and symptomatic events (18). Over the past five to 10 years, advanced technologies have emerged, targeting consumers and businesses. The most commonly used accelerometer is found in phones, wrists, and textiles. Accelerometers detect acceleration by measuring sensor displacement or mechanical stress applied in the system. They can also be used for heart sound monitoring by evaluating auditory vibrations, detecting conditions based on intensity, frequency, quality, and duration. However, noise from motion artifacts is a major limitation (19).

**Figure 2:**
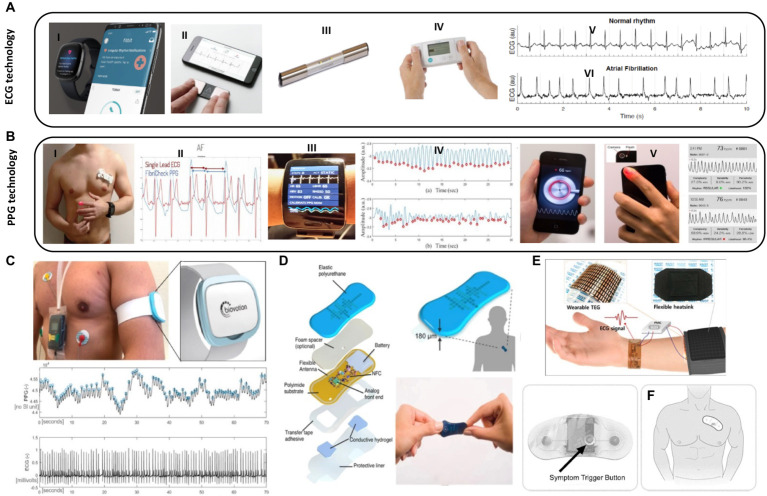
Wearable biosensors applications in cardiovascular care. **(A)** Examples of wearable tools used in clinical settings for ECG monitoring: (I) Fitbit-© Fitbit Inc., San Francisco, CA, United States, all rights reserved. (II) KardiaMobile-© all rights reserved (Mountainview, CA). (III) MyDiagnostick-© Applied Biomedical Systems BV, Maastricht, Netherlands, all rights reserved. (IV) Zenicor-ECG© Zenicor Medical Systems Ltd., London, United Kingdom, all rights reserved. (V) A sample of ECG graph for a healthy heart and, (VI) Afib ECG graph with irregular intervals. **(B)** Examples of wearable tools for PPG monitoring: (I) anatomical location of fibricheck patch on the chest-© Qompium, Hasselt, Belgium, all rights reserved. (II) Single lead ECG signal and fibricheck PPG signal. Reprinted from reference (25), licensed under CC BY 4.0. (III) Wristband of the Simband with LEDs for PPG and ECG. (IV) A sample 30 s clean PPG segment and corrupted PPG segment collected from Simband. Reprinted from reference (26), licensed under CC BY NC ND. (V) Smartphone camera‐based PPG measurements of the pulse waveform. Reprinted from reference (27), licensed under CC BY 4.0. **(C)** Recorded PPG and ECG signals with the medical wearable attached to the left upper arm vs. ECG Holter (Everion, Biovotion AG, Switzerland). Reprinted from reference (28), licensed under CC BY. **(D)** Schematic illustration and image of a soft flexible cardiac sensor in a thin elastic enclosure consisting of the multiple polymeric, electronic, adhesive and hydrogel layers and data transfer to smartphone app (via NFC) for visualization of logged heart rate data and/or real-time ECG waveforms. Reprinted from reference (29), licensed under CC BY. **(E)** Schematic diagram of a flexible self-powered wearable ECG system. Reprinted from reference (30), Copyright © 2018 American Chemical Society. **(F)** Zio patch button and placement-© iRhythm Technologies, Inc., San Francisco, United States, all rights reserved.

Ballistocardiogram (BCG) is an accelerometer-based technique that detects cardiac motion, allowing for the detection of heart rate, blood pressure, and myocardial contractility (20). Seismocardiogram (SDG) uses a chest wall sensor to detect chest wall vibrations, gathering information on heart rate, blood pressure, and cardiac output (21, 22). Photoplethymography (PPG) detects variations in blood pressure within microvasculature. It can be used with smartphones or accessory devices to measure pulsatile activity, detecting heart rate and heart rhythm, but measurements can be affected by body movement, temperature, hair, skin color, and tattoos (23, 24). Electrode-based technology is increasingly used in healthcare monitoring, recording single-lead electrocardiograph (ECG) using two vectors. These techniques can detect heart rate and rhythm, as well as ischemia, but these readings are subject to noise and artifacts and are limited to a single lead analysis. There are already several commercialized wearable ECG devices in the market including smartwatches, such as those offered by companies like Apple, Fitbit, and Samsung, often include ECG functionality as a feature (Figures 2A,B). These devices can measure heart rate and record ECG waveforms, providing users with insights into their cardiac health (31). Smart wristbands and patches are also available in the market, offering ECG monitoring capabilities. These devices are designed to be worn on the wrist or attached to the body, allowing continuous ECG monitoring, and other wearable devices, such as intelligent three-lead electrocardiograph monitors, have been developed to provide ECG monitoring with feedback functions for warning potential heart attacks (32). Patches with embedded electrodes have the ability to wirelessly transmit and record ambulatory ECG data for longer periods of time than a standard Holter. They offer details on the heart rate, pauses, high grade atrioventricular block, counts of isolated supraventricular and ventricular ectopic beats, and runs of supraventricular and ventricular tachycardia (Table 1). The Zio Patch (iRhythm Technologies, Inc. San Francisco, United States), a leadless electrocardiographic monitoring device, has been evaluated in 26,751 consecutive patients for its effectiveness in detecting arrhythmias. The findings could have significant implications for device selection, monitoring duration, and care pathways for arrhythmia evaluation and AF surveillance (33). ePatch is a lightweight, body-worn sensor that records and stores heart rhythm data, which can be downloaded and evaluated by cardiac monitoring professionals (34). A novel study evaluated the performance of a medical wearable, Everion^®^ (Biovotion AG, Switzerland), using passive PPG technology for AF detection in patients with paroxysmal or persistent AF during inpatient conditions (Figure 2C). Reliable and continuous monitoring of AF by employing a deep neural network demonstrated a 95.2% sensitivity and a 92.5% sensitivity (28). A prospective clinical trial compared the performance of a 14-day continuous electrocardiogram patch (EZYPRO^®^, Sigknow Biomedical Co., Ltd., Taipei, Taiwan) for detecting arrhythmias compared to conventional 24 h monitoring. The patch was associated with higher detection rates in patients with SVT, irregular SVT without P wave, AF/AFL, and critical arrhythmias (35). The KoMaWo configuration (SmartMedics, Poland), a new variation of ECG electrode positioning, has been tested on 15 patients with ST segment deviations due to coronary artery disease, offering extended monitoring and increased diagnostic accuracy for cardiac arrhythmias, making them a viable alternative to traditional cardiac monitoring methods (36).

**Table 1:** Various device types of wearable biosensors for cardiac function measurements.

Device type	Manufacturer	Product name	Cardiac function measurements	Other measurements	Official website
Watch	Apple	Apple Watch series7	HR, ECG, BP	SpO_2_, fitness tracking, health monitoring features	https://www.apple.com/
Watch	Galaxy	Galaxy Watch Active 2	HR, ECG, BP	Spo2, activity tracking, stress management, Health monitoring features	https://www.samsung.com/
Watch	Garmin	Garmin Venu 2/2S	HR	Blood oxygen levels measuring	https://www.garmin.com/
Watch	Fitbit	Fitbit Sense	HR, ECG, HR variability	Stress management, activity tracking, Afib assessment	https://www.fitbit.com/
Watch	WITHINGS	Withings ScanWatch	HR, ECG, BP	SpO_2_, sleep tracking, activity tracking, breathing disturbances tracking	https://www.withings.com/
Watch	Amazfit	Amazfit GTR 3	HR	SpO_2_, health and activity tracking, sleep monitoring	https://www.amazfit.com/
Watch	POLAR	Polar Vantage V2	HR	Fitness and health tracking, Recovery tracking	https://www.polar.com/
Wristband	Fitbit	Fitbit Charge 5	HR, ECG, HR variability	Health and fitness, sleep tracking	https://www.fitbit.com/
Wristband	Garmin	Vivosmart 4	HR	Smart fitness tracking, blood oxygen saturation, energy monitoring	https://www.garmin.com/
Wristband	WHOOP	WHOOP Strap 4.0	HR, HR variability	Overall cardiac function, guidance for workouts and recovery, sleep tracking	https://www.whoop.com/
Wristband	Amazfit	Amazfit Band 6	HR, HR variability	Health and fitness, sleep tracking, blood oxygen measurement	https://www.amazfit.com/
Wristband	Samsung	Samsung Galaxy Fit 2	HR	Activity and fitness tracking, sleep tracking	https://www.samsung.com/
Wristband	POLAR	Polar Ignite 2	HR	Sleep tracking, guidance for workouts and recovery	https://www.polar.com/en/ignite2
Wristband	WITHINGS	Withings Move ECG	HR, ECG	Activity and sleep tracking, early detection AFib	https://www.withings.com/
Chest strap	Qardio	QardioCore	HR, ECG, HR variability	Fitness tracking, skin temperature, respiratory rate	https://www.qardio.com/
Patch	VIVALINK	VivaLNK ePatch	HR, ECG, HR variability	Respiratory rate, skin temperature, step count, posture, 3-axis accelerometer	https://www.vivalink.com/
Patch	BioTel Heart	BioSticker	HR, ECG		
Patch	SmartCardia	SmartCardia 7L Patch	HR, ECG	Continuous ambulatory monitoring, managing arrhythmias	https://www.smartcardia.com/
Patch	NUUBO	NUUBO Smart Patch	HR, ECG	Arrhythmia events detecting like atrial fibrillation, tachycardia, bradycardia and others	https://www.nuubo.com/
Patch	Sigknow Biomedical Co.	EZYPRO	ECG	Arrhythmia detection, stroke prevention (ischemic/secondary), assess the cause of syncope (psychogenic), routine health check-up (early detection and timely treatment), post heart surgery monitoring	https://sigknow.com/en/ezypro/
Patch	iRhythm Technologies	Zio Monitor	HR, ECG	Detecting different types of arrhythmias, plus sinus rhythm and artifacts	https://www.irhythmtech.com/
Smart accessory	WITHINGS	BPM Core	BP, EKG	Advanced cardiac health monitoring, detecting masked hypertension, and manage nocturnal hypertension	https://www.withings.com/
Smart accessory	AliveCor	KardiaMobile	HR, EKG	Detect atrial fibrillation, Bradycardia, and Tachycardia	https://alivecor.com/
Smart accessory	OMRON Healthcare	Omron HeartGuide	BP	Daily activity tracking, sleep tracking, providing a comprehensive view of heart health	https://omronhealthcare.com/
Smart accessory	Empatica	Embrace2	HR	Electrodermal activity, providing insights into stress, detecting possible convulsive seizures	https://www.empatica.com/embrace2/
Smart accessory	Pulseon	PulseOn HR	HR, ECG	Asymptomatic arrhythmias detection, long-term monitoring of arrhythmias, e.g., atrial fibrillation (AF)	www.Pulseon.com
Smart accessory	Bittium	Bittium Faros 360	HR, ECG	Sports and medical monitoring, intelligent arrythmia detection	https://www.bittium.com/
Smart accessory	Komodo Tec	AIO Smart Sleeve	HR variability, ECG, EKG	SpO_2_, respiration rate monitoring, activity intensity	https://komodotec.com/product/aio-sleeve/
Smart accessory	Oura	Oura ring	HR	SpO_2_, skin temperature, sleep tracking	https://ouraring.com
Smart accessory	toSense	CoVa 2	HR, HR variability, ECG, strokevolume, cardiac output	Chest fluids, respiratory rate	https://www.tosense.com

Numerous devices are available for health monitoring, including the Apple Watch (Apple Inc., Cupertino, CA, United States), which incorporates accelerometer and PPG-based technologies for heart rate tracking and arrhythmia detection. Smartphone accessories like KardiaMobile (Mountainview, CA), Preventicus (Preventicus^®^, Jena, Germany), and Pulse-Smart (Medicore Co., Ltd. in South Korea) use PPG technology to detect arrhythmias and heart rate using touchpad electrodes (37). The Cardiio Rhythm smartphone PPG application (Cardiio Inc., Cambridge, United States) has provided an accurate and reliable means to detect AF in patients at risk of developing AF, potentially enabling population-based screening for AF (38). Other accurate blood pressure monitoring devices include a small sensor attached to a smartphone, a pressure sensor worn continuously over the wrist, and a system that incorporates both SCG and BCG technology through a chest patch and wrist watch (39). A vest with built-in sensors, electrode connections, and an optical probe clipped on the ear can monitor heart rate, oxygen saturation, and activity levels, as well as dynamic changes in cardiopulmonary function during activity (40). The majority of consumer electronics are heart rate or activity monitors, which can reduce the risk of cardiovascular disease. Wearing pedometers increases daily activity levels, decreases BMI, and lowers blood pressure (41). Mobile apps and wearable devices have been shown to lead to greater weight loss in obese patients (42). In addition, there are several other examples of intermittent atrial fibrillation detection tools used in clinical and research settings including MyDiagnostick (Applied Biomedical Systems BV, Maastricht, Netherlands), Zenicor-ECG, and FibriCheck (Qompium, Hasselt, Belgium). The MyDiagnostick and Zenicor-ECG are commercially available single-lead electrocardiograph devices for AF detection with high sensitivity and specificity (Figure A) (43, 44). FibriCheck is an FDA-cleared photoplethysmography-based smartphone application for heart rate measurement and AF identification (25).

Wearable devices are widely used for disease screening, particularly in detecting arrhythmias. The Apple Heart Study found that the Apple Watch can detect asymptomatic atrial fibrillation (Afib) using PPG technology (45). These devices can also help in risk stratification patients with known cardiovascular diseases by tracking vitals and activity levels. These devices significantly impact decision-making and help patients make informed decisions such as Galaxy Watch Active 2, Simband, and AliveCor’s KardiaBand (Figure 2D) (26, 27). In addition to disease prevention and screening, these technologies can also help in change management. For instance, a patient experienced ST depressions on their Apple Watch, which can alert them to arrhythmias and ischemia (46). Electrode technology has shown promise in detecting ischemia, as demonstrated in a study involving 200 patients with suspected STEMI symptoms. The study used smartphone-derived ECGs, Livecore, attached to an iPod Touch, and found a good correlation between the two. Overall, ischemia detection using Livecore can be relatively reliable (47). Another study involving 45 patients with chronic heart failure found that seismocardiography chest patch technology can differentiate between compensated and decompensated heart failure states. Although not widely used, these technologies have great potential in providing better care for patients (48).

Research on using wearable devices for activity tracking has been used in studies to determine the relationship between post-operative activity and length of stay after major surgeries, including cardiac surgery. It can also serve as an endpoint in drug trials, such as nitrates for heart failure patients to improve activity (49, 50). A meta-analysis of digital health interventions, including telemedicine, web-based monitoring, email messaging, mobile phone tracking, text messaging, and monitoring sensors, found that overall outcomes were better in patients who underwent digital health interventions compared to those receiving usual care. Out of the 1,200 patients in the digital health interventions group, about 100 developed events, while 160 in the usual care group developed events (51). Digital health interventions appear to have more effect on secondary prevention of cardiovascular diseases and heart failure patients compared to primary prevention. A pilot study showed that the ReDS vest may reduce heart failure readmissions. Fifty patients were admitted for decompensated heart failure and were instructed to wear the vest for 90 days. After 90 days, the readmission rates were compared. The vest significantly reduced heart failure readmissions compared to pre-and post-wear periods. Although not routinely used, the vest could significantly reduce the disease burden and mortality of cardiovascular diseases (52). With the emergence of the Internet of Things (IoT) era, the development of self-powered wearable medical sensors using flexible electronic devices is on-demand. A self-powered wearable electrocardiography (ECG) system was demonstrated, powered by a wearable thermoelectric generator (w-TEG) using body heat. Parametric studies were conducted on the PHS, and the w-TEG structure was optimized. The output power density was over 38 μW/cm for the first 10 min and 13 μW/cm for 22 h (Figures 2E,F) (29, 30).

## Wearable/implantable biosensors in neurological care

By providing valuable insights into the brain’s activities and overall health, wearable biosensors have the potential to enhance diagnostics, improve treatment outcomes, and enable personalized care for individuals with neurological conditions (53). In this section, we will explore the significant role of wearable biosensors in neurological care and their potential to transform the way we understand and manage neurological disorders.

**Figure 3:**
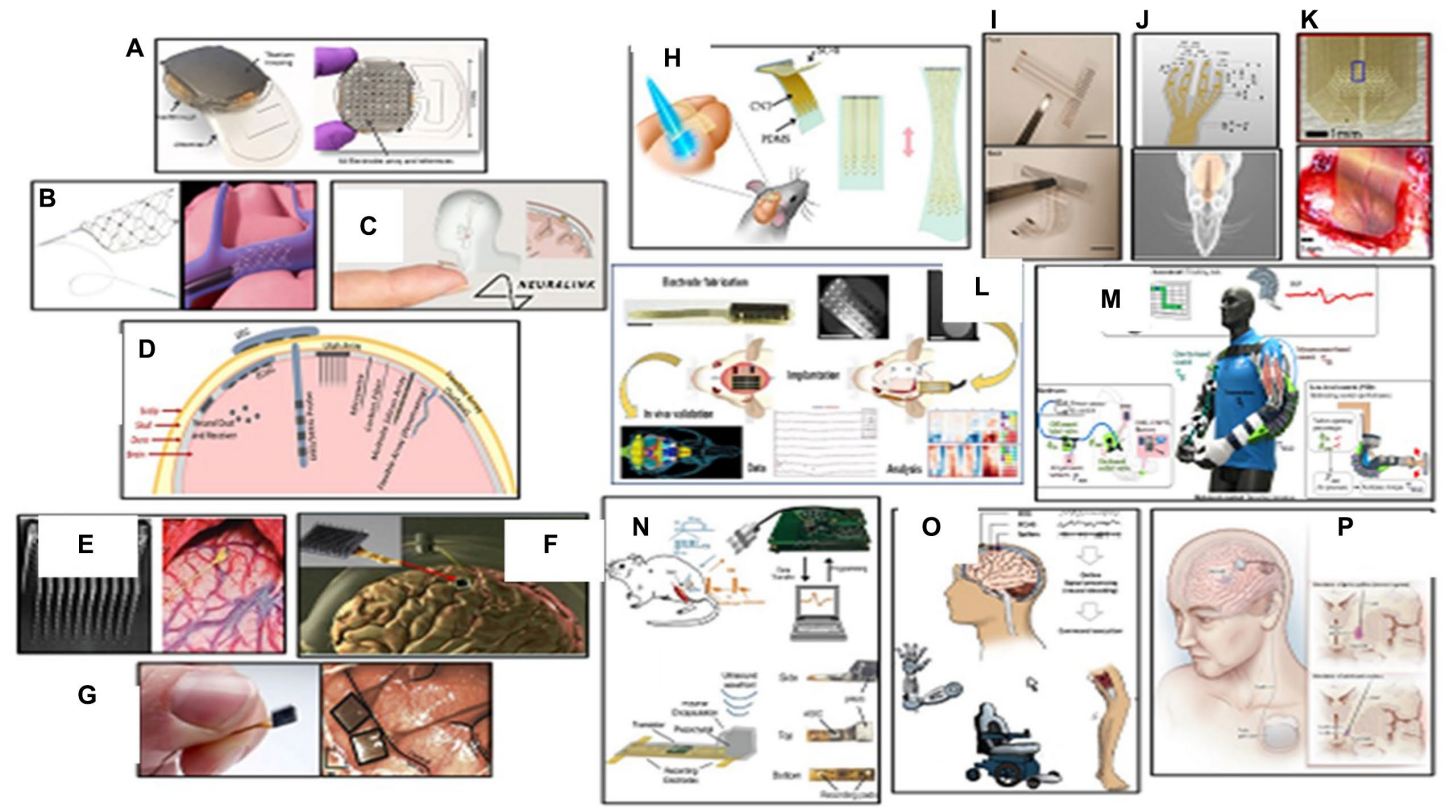
Wearable biosensors in neurological care. **(A)** Top and bottom view of WIMAGINE ECoG recording implant. Reprinted from reference (54), Copyright © 2015, IEEE. **(B)** Synchron’s Stentrode device expands inside a blood vessel on the brain to relay motor signals. Reprinted from reference (55), licensed under CC BY. **(C)** Neuralink implants are aimed at enabling human brains to communicate directly with computers. Reprinted from reference (56), © 2024 Springer Nature Limited. **(D)** Schematic view of the major types of BMI electrode interfaces in brain. Reprinted from reference (57), licensed under CC BY. **(E)** A 100 microelectrode Utah electrode array and the electrode probe tips implanted into the cortex of brain. Reprinted from reference (58), licensed under CC BY 4.0. **(F)** The BrainGate neurosensor composed of a silicon-based cortical microelectrode array implanted for intracortical neural microcircuit recording via a percutaneous connection to a skull mounted pedestal connector. Reprinted from reference (59), licensed under CC BY-NC-ND 4.0. **(G)** The NeuroPort Array chip used by Blackrock Neurotech in its devices. Reprinted from reference (55), licensed under CC BY. **(H)** Schematic view of a stretchable transparent electrode arrays for simultaneous electrical and optical interrogation of neural circuits *in vivo*. Reprinted from reference (60), Copyright © 2018 American Chemical Society. **(I)** Front view and backside of an inkjet printer thin flexible ECoG microelectrode array on a thin parylene-C film. Reprinted from reference (61), licensed under CC BY. **(J)** Schematic of a PDMS-parylene micro-electrode arrays (MEA) with 10 convex electrode sites in four arms for *in vivo* ECoG recording from rat olfactory bulb. Reprinted from reference (62), licensed under CC BY. **(K)** Photo of active area of a silicon carbide array that implanted on primary visual cortex of a rat for electrocorticography and peripheral nerve recording. Reprinted from reference (63), licensed under CC BY 3.0. **(L)** Electrode fabrication, implantation and *in vivo* validation of a flexible polymer microECoG array to map functional coherence in schizophrenia model. Reprinted from reference (64), Copyright © 2022, The Author(s) of (64), under exclusive licence to Springer Nature Limited. **(M)** A soft pneumatic exosuit consisting of two independent elbow sleeves with actuator tubes that snake through the posterior portion of the sleeves to provide flexion assistance. Reprinted from reference (65), licensed under CCD bY. **(N)** *In vivo* experimental setup for EMG recording using a neural dust mote was placed on the exposed muscle surface, the external transducer couple’s ultrasound to the mote, and the wireless data are recorded and displayed on the laptop. Reprinted from reference (66), © 2016 Elsevier Inc. **(O)** Brain-computer interfaces in neurorecovery and neurorehabilitation. Reprinted from reference (67), Copyright © 2021, Rights Managed by Georg Thieme Verlag KG Stuttgart • New York. **(P)** An illustration of deep brain stimulation for the treatment of Parkinson’s disease. Fine wires are implanted within brain regions involved in motor control, and stimulation is controlled by a pacemaker-like device under the skin. Reprinted from reference (68), Copyright © 2013, © 2013 Wolters Kluwer Health | Lippincott Williams.

The brain sends command prompts through the spinal cord, but the connection between the brain and the body can be broken due to physical injury or degenerative disease. The brain computer interface (BCI) can function as a bridge to bypass this broken connection. Existing BCI technology can be categorized into invasive and non-invasive (Figure 3A) (57). The Utah Array, the current industry standard, is a square computer chip with spikes that can read electrical signals from the cortex region of the brain. This process allows a person with a brain implant to control electronic devices with their brain, such as robotic limbs or computers. However, this method is limited to medical research environments (58). The next generation of BCI technology, led by startups like Synchron, Blackrock Neurotech, and Neuralink, could potentially become life-changing medical technology for paralyzed individuals in the near future. Blackrock’s device is implanted directly into the brain (Figure 3C), while Synchron’s is implanted into blood vessels in the brain (Figure 3B). Blackrock Neurotech’s BCI the NeuroPort, uses a series of tiny brain chips, called NeuroPort arrays which consist of 96 densely packed electrodes to record and stimulate neurons with high precision from virtually anywhere on the brain surface. Multiple arrays can be placed in one person, creating brain-computer interfaces and neuroprosthetics that can control objects and repair senses. The signals are transmitted wirelessly to an outside device like a wheelchair or cursor, giving people control over their environment (55). Blackrock has also partnered with the University of Pittsburgh’s rehab neural engineering labs to create the first portable brain computer interface, allowing patients to participate in research trials (69). Neuralink, a founding company, plans to implant electrodes directly into people’s brains, potentially providing a cure for neurological disorders like spinal cord injury, seizures, paralysis, and depression.

The Neuralink device, resembling a small coin, uses a robotic sewing machine to insert fine and flexible electrode wires into the outer cortex layer. This device could be used to operate robots, cure paralysis, treat mental illness, stream music, and extend hearing range beyond normal frequencies. The Neuralink device resembles the Utah array and could potentially cure neurological disorders (56). In mid-2021, ClearPoint Neuro and Blackrock Neurotech collaborated to develop an automated surgical solution for placing brain computer interface devices in patients with neurological disorders like paralysis, ALS, and hearing loss (Figure 3D) (67). Additionally, ClearPoint has partnered with Higgs boson health to launch a digital patient-facing application using the Manage My Surgery platform, focusing on drug delivery to the spine and brain, as well as BCI technology (Figure 3E) (70). BrainGate researchers have demonstrated the first human use of a high bandwidth wireless brain computer interface with an external wireless transmitter, allowing users to operate external devices like computers and robotic arms with their minds. Initial clinical research demonstrated the system’s ability to intuitively control advanced prosthetic limbs and robotic devices, providing paralysis patients with simple control, powerful assistive movement, and communication devices. The ultimate goal is for people to take the device home and use it for daily activities. This research aims to improve independence, mobility, and safety for blind people (Figure 3F) (59).

Electrocorticography (ECoG) is a non-invasive procedure that uses electrodes placed directly on the brain, allowing for better and more precise signals. It has a higher spatial resolution of approximately 4 square millimeters, making analysis more complex. ECoG grids consist of small disk-like electrodes placed epidurally or subdurally, and the distance between electrodes can vary depending on the region of interest (71). Epilepsy patients with ECoG are often observed for several days or weeks to identify epileptogenic zones, providing a unique opportunity to study electric activity at high spatial and temporal resolution (72). Recent advances allow for long-term or chronic placement of ECoG sensors, enabling the development of ECoG-based brain computer interfaces and closed-loop approaches (73). ECoG can also be used to control prosthetics, recording movement planning signals from the motor cortex and translating them to actual movement of an artificial arm or hand (74). EEG or EMG can be used to control robots and smart devices, such as prosthetics. The Walk Again Project has demonstrated the use of EEG-controlled exosuits to help people walk again, using motor-imagery patterns as triggers to move the robot’s legs (Figure 3M) (65). Mind-controlled wheelchairs provide basic directional control using motor imagery, p300 signals, or population encoding (75). CTRL labs developed a wristband that accurately maps finger actuation and hand positioning using electromyographic signals and accelerometer data (76). Several companies are working on developing novel EEG based BCI electrodes interpreting neural activity using artificial intelligence, allowing users to control objects with their minds (77, 78). In a more recent study, a digital bridge between the brain and spinal cord was developed based on the brain-spine interface (BSI) implant to restore communication between the brain and the spinal cord, allowing individuals with chronic tetraplegia to walk naturally in community (79).

Traditional BCIs have often utilized rigid electrodes, which, while effective in capturing neural signals, have faced challenges in terms of long-term use and patient comfort. The transition to flexible electrodes marks a significant advancement, addressing these concerns and opening up new possibilities for interfacing with the brain, highlighting significant benefits including enhanced comfort and biocompatibility, improved signal quality, versatility in placement, and long-term stability. Several promising reports are published on the applications of flexible BCI systems in health-related fields such as neuroprosthetics, neurorehabilitation, and cognitive enhancement (Figures 3H–K) (60–63).

A cutting-edge development in the realm of BCI systems is the emergence of portable, wireless, weakly-invasive BCIs that not only push the frontiers of performance but also enhance user convenience and accessibility. The convergence of portability, wireless connectivity, and weakly-invasive design in BCIs heralds a new era of human-computer interaction. As these systems evolve, they promise to redefine our relationship with technology, offering not only enhanced capabilities but also a glimpse into the profound possibilities of the human mind (Figure 2A) (54, 66, 80).

In 2013, Nicolelis and a team of researchers developed the first brain-to-brain interface (BTBI), enabling lab rodents to share sensorimeter information and work together for rewards (81). The BraiNet project connected the brains of three primates, allowing them to move an arm over a target in digital 3D space (82). BCI applications focus on total levels of brain activity and frequency bands, and are used for meditation, focus, and sleep enhancement. Neurofeedback technology changes cues based on brain indicators, such as wakefullness, and is used for meditation, sleep, focus, and seizure monitoring (83). A team of researchers developed a way for disabled artists to create art using a brain computer interface (84). The Neuracle NeuSenW is a fast, small, lightweight, compact, flexible, and wearable system that can transmit up to 64 channels of high-quality EEG wirelessly at up to 16 kilohertz per channel. This system is robust for ambulatory use in naturalistic environments and allows high-accuracy synchronization across multiple devices (85). BIOSEMI, Cognionics, ANT Neuro, G. Tec, Brain Products, and EMOTIV are among the most common EEG-based electrodes mobile systems used to study the neural control of human locomotion (86). A speech neuroprosthetic device was developed by researchers at the University of California San Francisco, which can decode full words and sentences from brain signals of participants who have been unable to speak due to a trauma-induced stroke. The device is placed over motor areas associated with speech and can decipher between words in a predetermined set and construct sentences. Data was collected from an electrocorticography array, semi-invasive, paper-thin grids of flat, circular electrodes placed underneath the skull but on top of the brain (87).

Parkinson’s disease (PD) is the second most prevalent neurodegenerative disease, affecting millions worldwide. Diagnosing PD can be challenging due to its diverse symptoms and similarities with other illnesses (88). Wearable brain implants have gained attention as valuable tools for managing PD, providing continuous monitoring and objective measurements of motor symptoms. Sensor-equipped wearable devices have shown significant potential in improving early diagnosis and monitoring of PD (89). One notable application of wearable brain implants in PD is the development of an Internet of Things (IoT) platform, pioneered by the Michael J. Fox Foundation for Parkinson’s Disease Research and Intel Corporation (90). Exopulse researchers have developed a suit called Mollii, which uses neuromuscular electrical stimulation to correct muscle tremors caused by degenerative brain disorders. This technology can effectively treat conditions like Parkinson’s, Cerebral Palsy, Spasticity, Multiple Sclerosis, and Chronic Pain (91). Deep brain stimulation (DBS) is FDA-approved for Parkinson’s disease and epilepsy, and has a humanitarian device exemption for dystonia and obsessive-compulsive disorder. There are three FDA-approved DBS therapy devices available: Medtronic, Abot SJM, and Boston Scientific. DBS has been used to treat chronic pain and depression, OCD, as well as other psychiatric disorders like anxiety and PTSD. DBS is like a pacemaker for the brain, improving quality of life by correcting abnormal brain rhythms (Figure 3P) (68, 92). Researchers are developing medical devices and AI-driven solutions to improve life for people with Parkinson’s. One such device is the CUE1 non-invasive wearable device, which uses pulsed cueing and focused vibrotactile stimulation to reduce symptoms of slowness and stiffness, resulting in improved movement (93).

The next generation of neural implants are being developed at Lawrence Livermore National Laboratory (LLNL) to deepen the exploration of the brain. In collaboration with the National Institute of Health (NIH), University of California San Francisco (UCSF), and other research institutes, engineers are developing brain-based healthcare devices using thin-film micro-electrocorticography surface arrays. These devices can record more information about an individual’s brain state, making treatment more informative and effective (94, 95). This technology is also paving the way for next-generation neural prostheses or implantable devices that could improve the ability of those with disabilities to see, speak, or hear (96, 97). Researchers have developed ultraflexible electrode arrays that can record thousands of neurons in live animals’ brains for months. These recordings enhance decoding accuracy during optogenetic stimulation and enable the detection of strongly coupled neuron pairs, enabling the study of large-scale neural circuits and patterns of information flow (Figure 3L) (64). UC Berkeley engineers have developed a wireless, implantable sensor that records electrical signals in nerves, providing real-time data for quadriplegic individuals to use prosthetic limbs. The neural dust used in the device records electrical activity in brain nerve cells, which can be analyzed to guide prosthetics (Figure 3N) (66). Engineers are working on creating a lifetime neural dust implant, which could potentially guide prosthetics and improve the quality of life for individuals with disabilities (98). Wearable biosensors are crucial in neurorehabilitation by enabling continuous monitoring and objective assessment of motor functions and movement patterns. These sensors promote adherence to therapy, monitor progress, and facilitate early intervention, contributing to neuroplasticity and functional recovery. Integrating wearable biosensors in neurological care fosters research and advancements, enabling population-scale studies to identify patterns, risk factors, and potential interventions for neurological disorders (99).

## Wearable/implantable wearable biosensors in ocular diseases

Ocular wearable contact lenses are a promising technology for non-invasive point-of-care testing and monitoring of various ocular diseases. These lenses make direct contact with ocular surfaces and are integrated with electronic devices and biosensors to detect biomarkers within the eye. These devices offer continuous and long-term measurement capabilities, enabling patients to manage their symptoms effectively and conveniently (100). The eye, being a complex sensory organ, contains abundant information that can be harnessed for wearable healthcare platforms. Ocular wearable devices, such as smart contact lenses or glasses, are designed to integrate biosensors that can measure various parameters related to ocular health. These biosensors can detect and monitor biomarkers in tears, enabling the assessment of physiological and pathological conditions (101).

Tears provide a direct connection to the blood and exhibit close correlations between tear and blood biomarker concentrations, allowing for the analysis of tear fluid as a non-invasive means of evaluating ocular health. Ocular wearable biosensors can detect specific biomolecules, such as proteins, enzymes, and metabolites, in tears, providing insights into ocular disease states. The analysis of tears offers potential for diagnosing ocular diseases, including conditions such as dry eye syndrome, glaucoma, and ocular inflammation (102).

Smart contact lenses offer noninvasive real-time detection of the human body for biomedical information. However, accurate measurement of physiological signals in tears is challenging. A self-powered multiplexed sensor based on organic electrochemical transistors (OSCs) was demonstrated, allowing semilog-linear response to glucose and calcium ions in tear fluids (103). Another interesting study represented a fluorescent scleral lens sensor was also developed based on a handheld ophthalmic readout device and a smartphone camera for quantitative measurements of physiological levels of pH, Na^+^, K^+^, Ca^2+^, Mg^2+^, and Zn^2+^ ions in point-of-care settings (104). A soft contact-lens biosensor (SCL-biosensor) was fabricated and tested for non-invasive biomonitoring of tear fluids, showing excellent correlation between output current and glucose concentration (105). A human pilot trial demonstrated that a novel soft, smart contact lens based on a graphene field-effect transistor sensor had a suitable sensitivity for real-time cortisol concentration detection in tears using a smartphone with a low detection limit (106).

**Figure 4:**
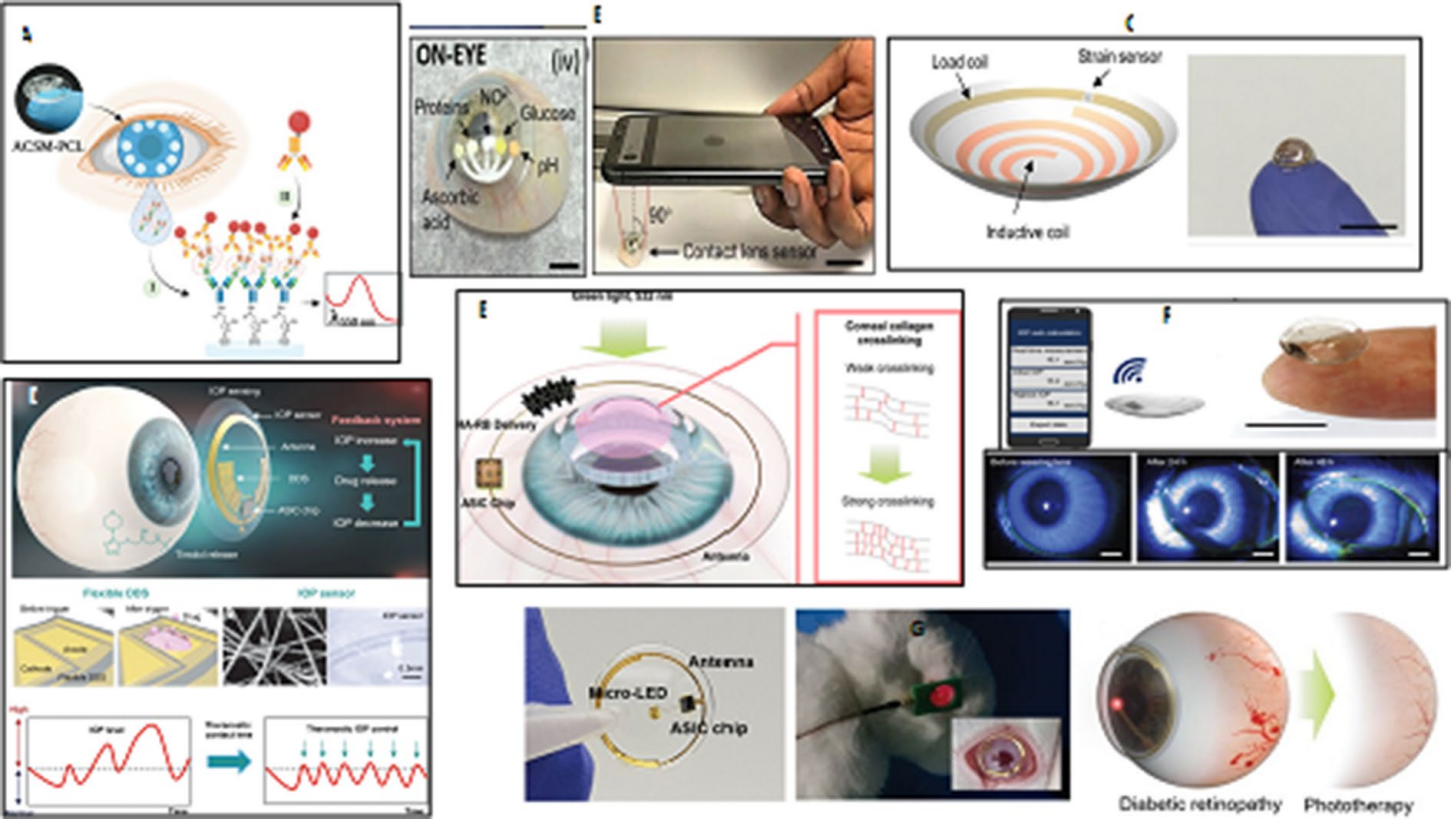
Ocular wearable or implantable biosensors. **(A)** An illustration of a PHEMA contact lens with embedded microchambers for the noninvasive detection of tear exosomes based on antibody conjugated signaling assay. Reprinted from reference (107), © 2022 Wiley-VCH GmbH. **(B)** The integration of paper microfluidics within laser-inscribed commercial contact lenses for multiplexed detection of clinically relevant analytes and diseases screening in the clinic or at the point-of-care. Reprinted from reference (108), © Royal Society of Chemistry 2020. **(C)** A soft, smart wireless contact lens for the real-time intraocular pressure monitoring following islet transplantation to the anterior chamber of the eye. Reprinted from reference (109), Copyright © 2020, American Chemical Society. **(D)** An illustration of a precisely integrated theranostic smart contact lens with a sensitive gold hollow nanowire based intraocular pressure sensor, a flexible drug delivery system, wireless power and communication systems and an application specific integrated circuit chip for both monitoring and control of intraocular pressure in glaucoma. Reprinted from reference (110), licensed under CC BY. 40. **(E)** A schematic view of a smart contact lens containing hyaluronate-rose bengal conjugate for biophotonic myopia vision correction. Reprinted from reference (111), Copyright © 2020, American Chemical Society. **(F)** A photograph of a soft and transparent contact lens for the wireless quantitative monitoring of raised intraocular pressure in real time using a smartphone. Reprinted from reference (112), Copyright © 2021, The Author(s) of (112), under exclusive licence to Springer Nature Limited. **(G)** Schematic illustration for the preparation and phototherapy of a smart wireless near-infrared light emitting contact lens for the treatment of diabetic retinopathy. Reprinted from reference (113), licensed under CC BY 4.0.

Recently, exosomes have gained attention as a valuable source of disease biomarkers. A poly (2-hydroxyethyl methacrylate) contact lens with antibody-conjugated signaling microchambers (ACSM-PCL) has been developed to detect tear exosomes that can detect exosomes in various solutions, including regular buffer, cell culture media, and human tears. The ACSM-PCL is expected to be a next-generation smart contact lens for cancer pre-screening and supportive diagnosis (Figure 4A) (107).

Several biochemical or biophysical sensors have been integrated into contact lenses for detecting single biomarkers in tear fluids, eyeball movement, and mechanical deformation. Wearable contact lenses monitor physiological parameters, but previous sensors only detect one analyte at a time. A multifunctional sensor was developed on an actual ocular contact lens using graphene and metal nanowires for measuring glucose levels in tears and intraocular pressure, evaluated in *in-vivo* and *in-vitro* tests using rabbit and bovine eyeballs. This system enables continuous, noninvasive monitoring of physiological conditions and biomarkers related to ocular and other diseases (114).

Microfluidics technology has been used to create contact lens sensors that can identify substances in tears, such as glucose, pH, nitrite, proteins, and ascorbic acid, through multiple sensing channels. A study demonstrated the integration of paper microfluidics in laser-inscribed contact lenses for multiplexed detection of clinically relevant analytes, such as hydrogen ions, proteins, glucose, nitrites, and L-ascorbic acid. This device has potential for medical diagnosis, disease screening, and monitoring of ocular infections, uveitis, diabetes, keratopathies, and oxidative stress (Figure 4B) (108). Microfluidic contact lenses have also been developed as wearable platforms for *in situ* tear pH, glucose, protein, and nitrite ions sensing. A microchannel was inscribed using CO_2_ laser ablation, and biosensors were embedded within microcavities that responded within 15 s, yielding high sensitivities in detection of pH, glucose, proteins, and nitrites. These contact lens sensing platforms could provide on-eye tears screening and monitor ocular health in clinics and point-of-care settings (115).

Evaporation, humidity, tears, and rain can affect eye concentration levels uncorrelated to blood concentration variation. Measurement in the tear film is insufficient for accurate electrochemical measurements and useful diagnostic conclusions. Electrochemical smart contact lenses (ESCL) have been developed to monitor chemical markers found in the tear film (116). A promising work presents a next-generation with four microelectrode arrays in lens quadrants, enabling real-time spatiotemporal sensing of concentration variation. The fast-switching chronoamperometric technique enables real-time electrochemical measurement of concentration flow, paving the way for clinical use (117).

Advancements in miniaturization, sensor technology, and wireless communication have led to the development of ocular wearable devices and biosensors. These devices allow for continuous monitoring of ocular health parameters, enabling early detection of ocular abnormalities and timely interventions. One significant application of contact lens sensors is the measurement of intraocular pressure (IOP), which is crucial for diagnosing and managing glaucoma (Figure 4F) (112, 118). Wearable contact lenses offer the potential for at-home monitoring without the need for patients to stay awake during measurements. Researchers have introduced integrated theranostic smart contact lens, which uses a gold hollow nanowire-based sensor, flexible drug delivery system, wireless power, communication, and an application-specific integrated circuit chip. This lens successfully monitors and controls pressure levels in glaucoma-induced rabbits, making it a promising personal healthcare platform for glaucoma and other ocular diseases (Figure 3D) (110). Several studies have been focused on developing functional contact lenses for regular tracking IOP because monitoring intraocular pressure (IOP) is crucial in the early diagnosis of glaucoma to prevent or slow down vision loss (119, 120). For example, a smart soft contact lenses (SSCL) for continuous 24 h monitoring of intraocular pressure (IOP) during sleep was introduced. These lenses retain intrinsic lens features like power, biocompatibility, softness, transparency, wettability, and oxygen transmissibility. They offer overnight wearability, ergonomic curvature fitting, mechanical and chemical durability, and disposable after multiple uses, making them crucial for transforming SSCL into glaucoma care (121).

Contact lens sensors have also been explored for the detection of other biomarkers and ocular pathologies. For example, the integration of biosensors with contact lenses enables the measurement of glucose levels in tear fluid, offering potential applications in diabetes management (122). These lenses also have the potential to detect various ocular conditions and optimize pharmaceutical treatments (123). Diabetic patients with poorly managed blood sugar levels are at risk of developing glaucoma, cataracts, and different degrees of diabetic retinopathy. Measuring tear glucose levels can be implemented as an alternative to traditional strips for noninvasive monitoring of blood glucose levels in diabetes mellitus diagnosis and treatment. A nanoparticle embedded contact lens (NECL) was developed as a biocompatible biosensor for noninvasive glucose monitoring, combining glucose oxidase and cerium oxide (III) and detecting changes in the reflection spectrum with glucose concentration (124). Furthermore, a noninvasive smart wireless far red/near-infrared (NIR) light emitting contact lens has been developed for repeated treatment of diabetic retinopathy. The lens uses a far red/NIR LED, an integrated circuit chip, wireless power, and communication systems, demonstrating its safety and feasibility in biomedical photonic applications. *In vitro* characterization confirmed that repeated wearing reduced retinal vascular hyper-permeability in rabbits (Figure 4G) (113). In recent years, intraocular islet transplantation has been introduced as a new diabetes treatment procedure that requires close monitoring of eye and islet graft function. A soft, smart contact lens has been developed to monitor intraocular pressure, detecting changes in pressure and transmitting real-time values wirelessly (Figure 4C) (109).

Revolutionize ophthalmology by providing valuable insights into ocular health, facilitating early diagnosis, and enabling personalized treatment strategies. The non-invasive nature of tear analysis and the convenience and accessibility of ocular wearables hold promise for improved ocular disease management and patient care. Advanced flexible contact lenses can dynamically monitor vital ocular indicators, spot abnormalities, and provide biofeedback guidance for real-time diagnosis and rehabilitation tracking of chronic eye diseases (125). A flexible multifunctional lens, based on inorganic γ-Fe_2_O_3_@NiO magnetic oxide nanosheets, has been designed with the capability to monitor glucose levels, eyeball movement, and intraocular pressure, offering personalized and efficient health management (114). Researchers have developed 2D biocompatible, flexible plasmonic contact lenses for red-green color blindness, which are low-cost, simple, and based on soft nano-lithography. These lenses offer new insights for color blindness correction applications due to their biocompatibility, low cost, stability, and simplicity (126). A pilot trial of a smart contact lens and skin-attachable therapeutic device for wireless monitoring and therapy of chronic ocular surface inflammation (OSI) has been conducted. The smart contact lens measures matrix metalloproteinase-9 concentration in tears, while the therapeutic device is a stretchable heat patch (127). Both devices can be integrated with smartphones for wireless communication, enabling instant diagnosis and automated hyperthermia treatments. *In vivo* tests confirm their biocompatibility and reliability as a noninvasive, mobile health care solution.

The materials used in contact lens sensors play a crucial role in their performance. Biocompatible polymers, such as hydrogel silicones, are commonly used for constructing the lenses, ensuring comfort and safety during prolonged wear (128). Lenses are integrated with biosensors that can measure specific analytes in tear fluid, ocular surface temperature, intraocular pressure (IOP), pH value, and other relevant parameters. To address the keratoconus, myopia, and corneal deformation, a biocompatible dye, RB, was conjugated to hyaluronic acid (HA) to enhance corneal permeability. This smart contact lens have potential on-demand HA-RB conjugate delivery, potentially enabling biophotonic myopia vision correction (Figure 4E) (111). In another attempt, an LC resonator strain sensor in a contact lens composed of a stretchable inductance coil and a chip capacitor has been developed for real-time IOP monitoring. It is ultra-soft, comfortable, safe, and stable, with linear and stable responses. It has been calibrated on silicone rubber eyeballs and has a higher sensitivity than mainstream lens sensors, making it a promising approach for 24 h continuous IOP monitoring in clinics (119).

As a multifunctional platform, contact lenses can simultaneously serve as wearable sensors for continuous monitoring ocular diseases and as a drug delivery system for treating these diseases. Soft contact lenses have the potential to deliver a variety of drugs, including antibiotics, antifungals, anti-inflammatory agents, and even glaucoma medications. The drug delivery system is achieved through the incorporation of the drug into the lens material or using the lens as a reservoir to release the drug over time. Microfluidic and electrochemical contact lenses offer the potential for personalized medicine and individualized drug regimens, allowing healthcare professionals to customize the drug release profile based on the specific needs of each patient. This personalized approach enhances treatment outcomes while minimizing the risk of drug resistance and optimizing therapeutic efficacy (129).

## Wearable/implantable wearable biosensors in continuous glucose monitoring

**Figure 5:**
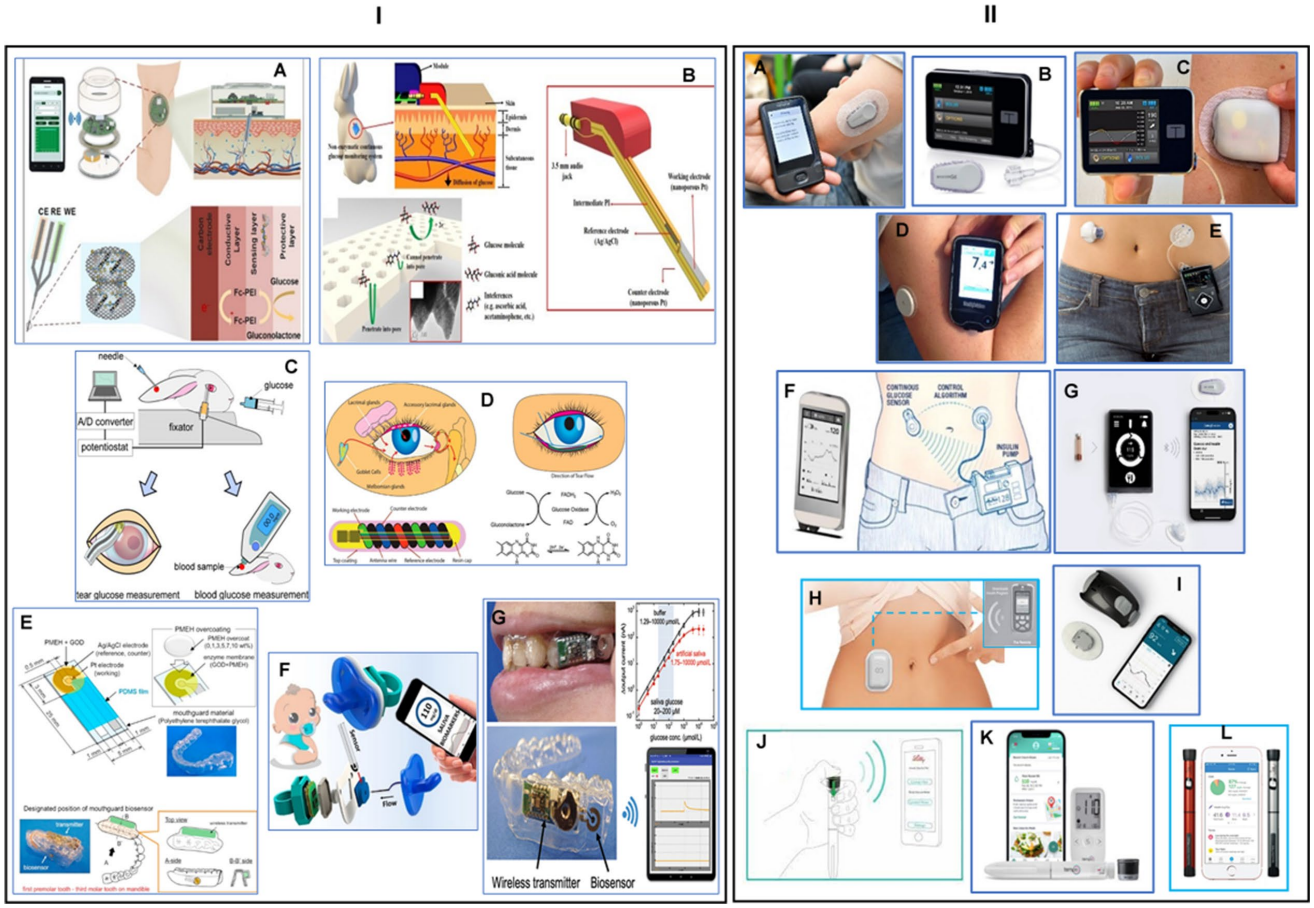
Wearable or implantable biosensors for continuous glucose monitoring. (I) Non-invasive wearable or implantable glucose monitoring systems: **(A)** an illustration of a wearable, robust, flexible, and non-enzymatic continuous glucose monitoring system, implanted into subcutaneous tissue for measuring interstitial fluid (ISF) glucose. The developed wireless system included electrochemical analysis circuits, a microcontroller unit, and a wireless communication module. Reprinted from reference (130), © 2018 Elsevier B.V. All rights reserved. **(B)** Schematic view of a smartphone-controlled and microneedle (MN)-based wearable CGM system for long-term glucose monitoring and home-care diabetes management. Reprinted from reference (131), Copyright © 2023, American Chemical Society. **(C)** Development of a soft contact lens biosensor for *in situ* monitoring of tear glucose as non-invasive blood sugar assessment. Reprinted from reference (132), Copyright © 2010 Elsevier B.V. All rights reserved. **(D)** Schematic illustration and properties of NovioSense minimally-invasive tear glucose sensor as an alternative to painful finger-prick for diabetes management utilizing a biopolymer coating. Reprinted from reference (133), licensed under CC-BY-NC-ND. **(E)** Designing a mouthguard biosensor as a novel cavitas sensor with telemetry system for monitoring of saliva glucose. Reprinted from reference (134), © 2015 Elsevier B.V. All rights reserved. **(F)** A fully integrated pacifier operating as a portable wireless device toward noninvasive chemical monitoring including glucose in the infant’s saliva. Reprinted from reference (135), Copyright © 2019, American Chemical Society. **(G)** A wearable cellulose acetate-coated mouthguard biosensor as a useful method for the unrestricted and noninvasive monitoring of saliva glucose for the management of diabetes patients. Reprinted from reference (136), Copyright © 2020 American Chemical Society. (II) Real-time continuous glucose monitoring systems: **(A)** a photograph of a Dexcom G6 real-time system-© Dexcom, Inc. San Diego, CA, United States, all rights reserved; reference (137). **(B)** Tandem t: slim X2 insulin pump-© Tandem Diabetes Care, Inc., San Diego, CA, United States, all rights reserved. **(C)** Omnipod 5 automated insulin delivery system-© Insulet Corp., Bedford, MA, United States, all rights reserved; reference (138). **(D)** Abbott’s freestyle libre glucose monitoring system-© Abbott Park, Illinois, United States, all rights reserved; reference (139). **(E)** Medtronic MiniMed insulin pump system-© Medtronic, Inc. Northridge, United States, all rights reserved; reference (140). **(F)** Artificial pancreas for automated blood glucose control-Copyright © 2017 Elsevier Ltd, reference (141). **(G)** The iLet beta bionics artificial pancreas system-Copyright © 2021 by the American Diabetes Association, reference (142). **(H)** Eopatch tubeless insulin pump-© EOFLOW Co., Ltd., Republic of Korea, all rights reserved; reference (143). **(I)** Waveform glucose monitoring technology-© WaveForm Technologies Inc. Wilsonville, OR, United States, all rights reserved; reference (144). **(J)** Smart insulin pen for personalized diabetes management-Copyright © 2023 American Diabetes Association, reference (145). **(K)** Tempo^™^ personalized diabetes management platform consisting of a pen, Smart Button, App, and a smartphone-© Lilly Corporate Ctr., Indiana, United States, all rights reserved; reference (146). **(L)** Novo nordisk smart pens for independently management of blood glucose values and prescribing regimen with support from the healthcare practitioner-© Novo Nordisk A/S, Bagsværd, Denmark, all rights reserved; reference (147).

In the dynamic landscape of healthcare, non-invasive glucose monitoring biosensors have emerged as beacons of progress, introducing a paradigm shift in the management of diabetes. Imagine a world where individuals no longer need to endure the inconvenience of routine finger-pricking, and instead, gain real-time insights into their glucose levels effortlessly. In this narrative of innovation, non-invasive glucose monitoring biosensors in tears, sweat, saliva, and interstitial fluid stand as pillars of progress, embodying the spirit of a future where healthcare is both personalized and seamlessly integrated into our daily lives. The amalgamation of data from tears, sweat, saliva, and interstitial fluid opens avenues for comprehensive health analytics. Researchers can harness this wealth of information to not only refine diabetes management strategies but also contribute to a broader understanding of the intricate interplay between glucose regulation and overall health. Non-invasive glucose monitoring biosensors have been meticulously designed to analyze specific biomarkers in saliva, offering a painless and convenient alternative to traditional blood-based methods. The richness of information within saliva provides a comprehensive view of glucose dynamics, enabling a more nuanced and personalized approach to diabetes management (Figures 5I, E–G) (134–136). Furthermore, interstitial fluid, the fluid that surrounds cells in our bodies, has become another focal point for these biosensors. By tapping into this reservoir just beneath the skin, biosensors can glean precise glucose readings without the need for invasive procedures. This approach not only enhances user comfort but also facilitates continuous monitoring, providing a continuous stream of data crucial for understanding glucose fluctuations throughout the day (Figures 5I, A,B) (130, 131). With advancements in biosensor technology, sweat has become a dynamic canvas for monitoring glucose levels. Wearable devices equipped with these biosensors now offer athletes, fitness enthusiasts, and individuals at large an unobtrusive means of tracking glucose fluctuations during physical activities, providing a valuable tool for optimizing performance and health (148). Tears are also used as a promising fluid for non-invasive glucose monitoring using wearable biosensors, providing a gentle and hassle-free means of assessing glucose levels that seamlessly integrating glucose monitoring into daily life without the need for intrusive measures (Figures 5I, C,D) (132, 133).

In the last 20 years, continuous glucose monitor (CGM) has significantly transformed the way diabetes is managed. CGM systems use a tiny sensor to test the interstitial fluid between body cells, checking glucose levels every 5 min. The information is then sent wirelessly to a reader, which displays the levels. Most CGM systems have three parts: a disposable sensor, a transmitter, and a monitor, typically smaller than a cell phone. This technology enables people with diabetes and healthcare professionals to monitor glucose readings on a receiving device or app, allowing for more accurate treatment decisions. Real-time CGM systems may reduce the need for regular finger pricks for individuals with diabetes (149). Sensor-based automatic insulin delivery devices have entered the market since the introduction of the first automatic insulin delivery device in 2006. These include Dexcom, Medtronic, Freestyle Libre, Eversense, Omnipod, T:slim X2, Tandem Mobi, Waveform cascade CGM, Bigfoot diabetes management system, Lily connected smart pen, and Novopen 6 smart pen. The Omnipod 5 pump device consists of a Bluetooth-enabled pod with insulin, a Dexcom G6 sensor that communicates directly with the pod, and a personal diabetes manager device that sends glucose values commands to the pod (Figure 5II, C) (138). The Dexcome G7 is a smaller, longer-weighted device that combines the sensor and transmitter in the same device (Figure 5II, A) (137). The Medtronic MiniMed 780G is an advanced hybrid closed loop system (AHCL) using a new generation 4 sensor, requiring only one calibration on the first day of wear (Figure 5II, A) (140). The Freestyle Libre 3 device is a thin real-time sensor that transmits data to the phone every minute and displays it on the mobile app (Figure 5II, D) (139). The Waveform cascade device is a 15-day CGM with a rechargeable transmitter approved in Europe in 2019, and communicates with a mobile app via Bluetooth with high accuracy (Figure 5II, I) (144). The Bigfoot Unity diabetes management system integrates a smart pen cap with the Freestyle Libre 3 to transmit insulin dosing information between the pen and mobile app (Figure 5II, J) (145). There are two versions of the pen cap, one for long-acting and the other for rapid-acting insulins. Lily’s disposable pen transmits data from the pen to the mobile app on the dosing and has a Tempo smart button. NovoPen 6 is another smart CGM pen made by Nova Nordis (Figure 5II, L) (147). The mylife YpsoPump is a small and light smart CGM sensor that uses an orbital infusion set, saving crimping and other problems (Figure 5II, K) (146). The EOpatch is a tubeless insulin patch pump developed by EOflow company, undergoing FDA approval. It is a small pod that stores insulin attached to the skin and can be used for 3.5 days or replaced on fixed days of the week for regular use. The closed-loop system mimics the human pancreas, automatically delivering the exact dose of insulin needed (Figure 5II, H) (143).

Over the past 15 years, scientists have been working on the artificial pancreas project, which aims to incorporate the essential function of the human pancreas. The bionic pancreas requires a smartphone as the command and control center housing, a software program that determines actions based on data from the glucometer, and a glucometer capable of continuous monitoring of blood glucose levels (150). A study by Russell et al. (151) compared the effectiveness of a Dexcom G4 platinum continuous monitor glucometer to an insulin pump, which accurately assesses blood glucose levels through a subcutaneously placed probe. The third component is a pump with separate reservoirs of insulin and glucagon, which can inject hormone subcutaneously into the user using a signal from the cellphone program. Patients with type 1 diabetes rely on various insulin delivery methods, such as insulin pens, insulin pumps, or hybrid closed-loop systems, which require substantial use interaction. The bionic pancreas is highly automated and requires only the patient’s body weight to start therapy. An impressive study, sponsored by the National Institute of Diabetes and Digestive and Kidney Diseases, demonstrated that automated glucose control with a bionic pancreas can significantly reduce the glycated hemoglobin level in children and adults with type 1 diabetes (Figure 5II, F) (141). The iLet bionic pancreas is the first fully automated insulin pump, requiring only weight and input from daily meals to learn insulin requirements. A study of over 400 participants found that after 3 months, the iLet insulin pump reduced HbA1c by 0.5%, increased time and range by 2.6 h, and did not increase hypoglycemia. The wearable device can be adjusted to function as an insulin-only, glucagon-only, or bi-hormonal artificial pancreas using both insulin and glucagon (Figure 5II, G) (142). Several successful trial studies have evaluated artificial pancreas for use by type 2 diabetes patients (152, 153). Cambridge scientists developed an artificial pancreas for type 2 diabetes patients using an algorithm developed at the University of Cambridge. The CamAPS FX device combines an off-the-shelf glucose monitor and insulin pump with an app that predicts insulin requirements. The device is fully closed loop, requiring no kidney dialysis, unlike the artificial pancreas used for type 1 diabetes. The Nature Medicine team reported the first trial of this fully closed-loop system for type 2 diabetes patients, recruiting 26 patients and dividing them into two groups. After 8 weeks, the average glucose levels fell from 12.6 mmol/L to 9.2 mmol/L while using the artificial pancreas. The app also reduced levels of glycated hemoglobin (HbA1c), which helps clinicians understand an individual’s average blood sugar levels over weeks or months (154).

## Wearable/implantable electrochemical sensors for non-invasive monitoring biometric signals

House monitors like Apple Watch and Fitbit primarily track health signs during physical activities, but they lack molecular information. This presents a significant challenge for developing wearable devices that track chemical biomarkers continuously and non-invasively. Electrochemical sensors offer high sensitivity, selectivity, speed, miniaturization, low costs, low energy consumption, and ease of use. These sensors offer numerous advantages, including continuous chemical information, better adherence to health, nutrition, and wellness assessment, self-care, preventive medicine, distance diagnosis, lower healthcare costs, and improved people’s lives (155). However, these sensors face challenges such as stability, accuracy, safety, compliance with body movement, energy demands, big data, and data security (156). There is a wide range of wearable electrochemical sensors for real-time continuous non-invasive monitoring of various substances, including metabolites, electrolytes, drugs, hormones, vitamins, cytokines, stress markers, and disease markers (157, 158). These sensors can be flexible and printable electrodes incorporated into clothing or attached directly to the body. Screen-printing technology, used for single-use disposable glucose biosensor strips, is an attractive route for fabricating wearable electrochemical sensors. Scientists worldwide are now using large-scale low-cost sensor fabrication to create a wide array of wearable sensor patterns on flexible substrates or textiles (159). However, the lack of stretchability of electrochemical devices hinders their wearable applications, and it is crucial to bridge the gap between softness of biology and rigid electrochemistry by developing flexible and stretchable printed electrochemical devices (160).

**Figure 6:**
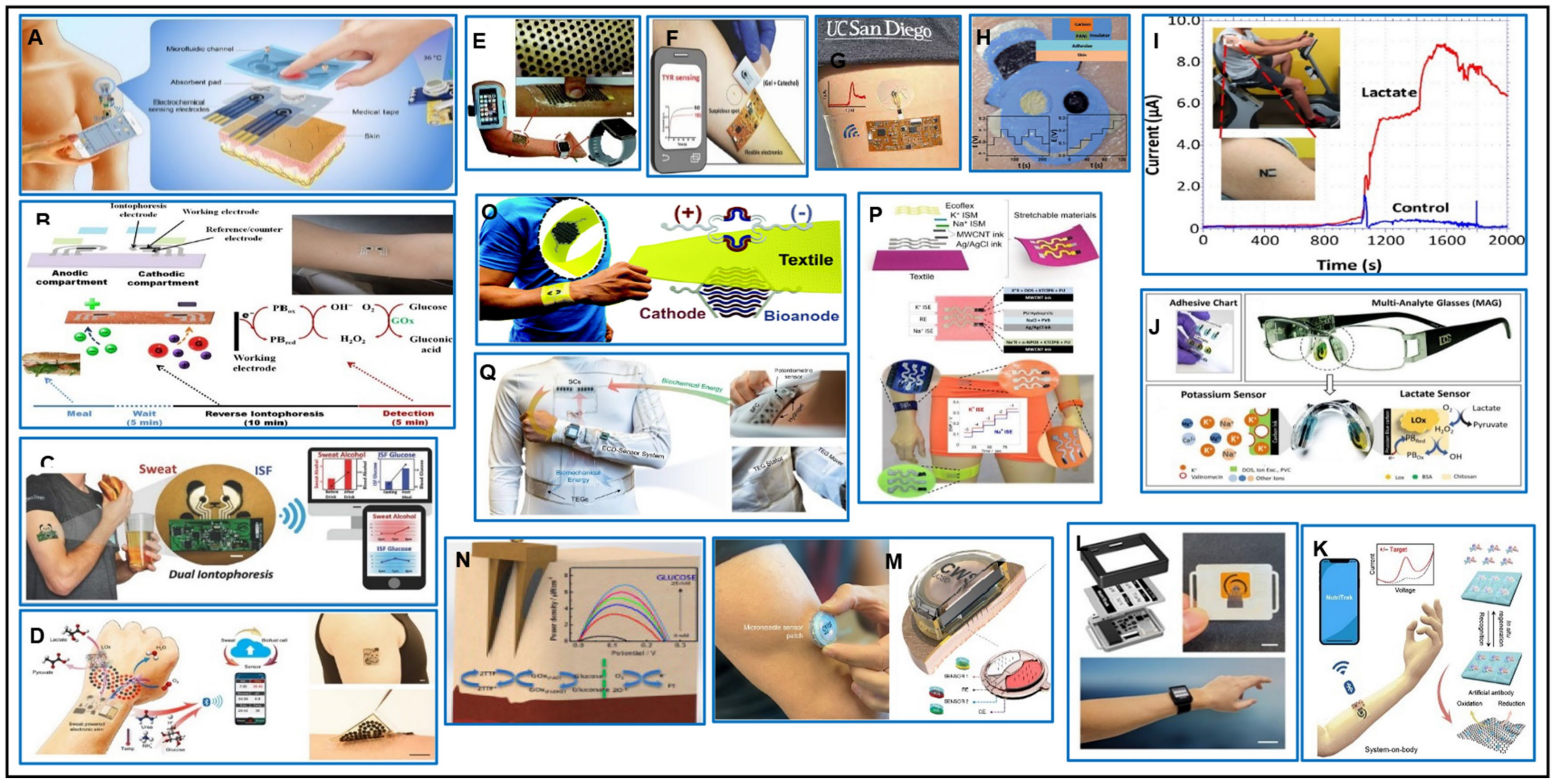
Wearable electrochemical biosensors. **(A)** Schematic view of a wireless, battery-free, flexible, self-pumping sweat-sensing system that simultaneously tracks levodopa and vitamin C levels in human sweat and detects body temperature. Adapted with permission from reference (164), Copyright © 2023, Tsinghua University Press. **(B)** Schematic of the printable iontophoretic-sensing system displaying the tattoo-based paper, Ag/AgCl electrodes, Prussian blue electrodes, transparent insulating layer, and hydrogel layer applied to a human subject. Reprinted from reference (165), Copyright © 2014 American Chemical Society. **(C)** Depiction of wearable iontophoretic biosensor device on a printed tattoo platform for glucohol sensing on a human subject, along with wireless real-time transmission of the ISF glucose and sweat alcohol response. Reprinted from reference (166), licensed under CC BY 4.0. **(D)** Schematic of a battery-free, biofuel-powered e-skin that efficiently harvests energy from the sweat, performs multiplexed biosensing, and wirelessly transmits data to a mobile user interface through Bluetooth. Reprinted from reference (167), Copyright © 2020 The Authors of (167), some rights reserved; exclusive licensee American Association for the Advancement of Science. **(E)** Illustration of a soft, stretchable electronic-skin-based biofuel cell, representing the highest power density recorded by a wearable biofuel cell, arranged in a stretchable “island-bridge” configuration. Reprinted from reference (168), © Royal Society of Chemistry 2017. **(F)** Representing of a wearable bendable bandage-based sensor and a minimally invasive microneedle electrochemical biosensor based on the presence of the tyrosinase (TYR) enzyme cancer biomarker toward rapid screening of skin melanoma. Reprinted from reference (169), © 2018 WILEY-VCH Verlag GmbH and Co. KGaA, Weinheim. **(G)** Describing a flexible epidermal microfluidic detection platform fabricated through hybridization of lithographic and screen-printed technologies, for efficient and fast sweat sampling and continuous, real-time electrochemical monitoring of glucose and lactate levels. Reprinted from reference (170), Copyright © 2017 American Chemical Society. **(H)** Presenting a novel tattoo-based solid-contact ion-selective electrodes (ISEs) for non-invasive potentiometric monitoring of epidermal pH levels. Reprinted from reference (171), © Royal Society of Chemistry 2013. **(I)** Presenting the first example of real-time noninvasive lactate sensing in human perspiration during exercise events using a flexible printed temporary-transfer tattoo electrochemical biosensor that conforms to the wearer’s skin. Reprinted from reference (172), Copyright © 2013 American Chemical Society. **(J)** Photograph of the eyeglasses biosensor system integrated with wireless circuit board and the nose pad electrochemical sensors for potassium and lactate sensing. Reprinted from reference (173), © Royal Society of Chemistry 2017. **(K)** Schematic of the wearable “NutriTrek” that enables metabolic monitoring through a microfluidic patch for sweat induction, sampling and biosensing. Reprinted from reference (174), Copyright © 2022, The Author(s) of (174), under exclusive licence to Springer Nature Limited. **(L)** “NutriTrek” smartwatch with a disposable sensor patch and an electrophoretic display. Reprinted from reference (174). **(M)** Illustrating of a fully integrated wearable array of microneedles for the wireless and continuous real-time sensing of two metabolites (lactate and glucose, or alcohol and glucose) in the interstitial fluid. Reprinted from reference (175), Copyright © 2022, The Author(s) of (175), under exclusive licence to Springer Nature Limited. **(N)** A schematic of the first example of microneedle-based self-powered biofuel-cell glucose sensor aimed at harvesting biochemical energy from the wearer’s transdermal fluid. Reprinted from reference (176), Copyright © 2014 Elsevier B.V. All rights reserved. **(O)** Demonstrating the first example of a stretchable and wearable textile-based hybrid supercapacitor-biofuel cell (SC-BFC) system, screen-printed on both sides of the fabric, scavenge biochemical energy from the wearer’s sweat. Reprinted from reference (177), © Royal Society of Chemistry 2018. **(P)** Image of the stretchable printed sensors on different common textiles and typical time trace plots for potassium and sodium. Reprinted from reference (178), © 2016 WILEY-VCH Verlag GmbH & Co. KGaA, Weinheim. **(Q)** Photographs illustrating the arrangement of the individual modules of the wearable microgrid system on a shirt worn on-body, including the TEG modules on the side of the torso, the SC modules on the chest, the BFC modules and potentiometric sensor inside the shirt for direct sweat contact, and wearable electronics that are powered by the microgrid. Reprinted from reference (179), licensed under CC BY 4.0.

Epidermal monitoring offers non-invasive ways to assess a wearer’s physiological state through lab-on-skin devices like E-skin tattoo biosensors (161), printable textile-based sensors (162), and minimally-invasive microneedle sensors (163). These devices integrate tattoo-transfer and thick-film fabrication processes to continuously monitor sweat chemistry (Figure 6H) (171, 172). The first epidermal enzyme electrode was designed to monitor sweat lactate concentration (Figure 6I) (172), and other flexible printable temporary-transferred tattoos functionalized with the enzymes were used for glucose and alcohol concentration in sweat or interstitial fluid (ISF) for fitness and performance (180). Researchers have developed a temporary skin-worn electrochemical biosensor for non-invasive glucose monitoring, combining reverse iontophoretic extraction of interstitial glucose with an enzyme-based amperometric biosensor. This device’s data is comparable to traditional blood glucose measurements (Figure 5B) (165). Researchers are now able to monitor simultaneously both sweat alcohol and glucose ISF by designing a single flexible wearable biosensor platform, enabling on-demand sampling and monitoring of both biofluids (Figure 6C) (166). These studies are moving towards personalized nutrition, providing timely nutrition feedback and guidance for supporting dietary behavior change. The ultimate goal towards personalized nutrition is to get comprehensive information about monitoring nutrition like metabolite, electrolyte, calcium, sodium, and potassium continuously at the molecular level not only in sweat but in tears, saliva, and ISF (181). Recently, researchers have developed wearable chemical-electrophysiological hybrid sensing systems that integrate multiple sensing modalities into a single platform. These systems record vital signs and chemical information simultaneously, such as blood pressure chemical sensing (BPCM) and electrochemical sensors for sweat and ISF biomarkers like lactate and glucose. The multimodal sensing without crosstalk, skin comfortability, and mechanical resiliency make it possible for performance during exercising and daily activities (Figures 6K,L) (174).

Fingertips are one of the sweatiest spots on the body and offer opportunity of sensing and energy applications without the need for sweat stimulation. Scientists have developed a touch-based technique for fingertips sweat bioelectronics, utilizing passive perspiration from the fingers without the need for sweat stimulation. This technique allows for rapid sensing of cortisol concentration in natural fingertip sweat, allowing label-free measurement from the decreased current response of the PB redox probe embedded in the polymeric network (182). The fingertip touch-based sensor was used in Parkinson’s patients to track L-dopa pharmacokinetic profile following oral tablet administration (183). A dual disposable fingertip touch-based sensing device was developed, integrating neighboring ketone and glucose enzyme electrodes on a single-strip substrate for simultaneous detection of ketone and glucose (184). Different epidermal sensing devices were designed in the form of fashion accessories like sunglasses, gloves, and bandages. The eyeglasses-based wireless sensor platform consists of potentiometric sensors for electrolytes and amperometric sensors for metabolites, allowing real-time monitoring of metabolites or electrolytes in tears and sweat (Figure 6I) (173).

Tattoo-based sensors have long mechanical strain capabilities, but recent studies focus on sweat microfluidic analysis for efficient sampling, flow, and removal. A skin-mounted microfluidic device, developed at UC San Diego University, is now commercialized by Innovosens for non-invasive, simultaneous multiparametric measurement of sweat metabolites for sport and fitness (Figure 6G) (170). Printable textile-based sensors with super mechanical stress-enduring features have been developed, embedding sensors directly on the elastic waist of underwear. Examples include a highly stretchable and printable textile-based potentiometric sensor array, which combines stretchable components like polyurethane, Ecoflex, and stretch-enduring inks for simultaneous multi-ion sweat analysis (Figure 6P) (178). Bandage-based biosensors are also well-performance wearable electrochemical sensors for monitoring wound healing and detecting the presence of tyrosinase (TYR) enzyme cancer biomarker in the presence of its immobilized catechol substrate for melanoma cancer screening and diagnosis (Figure 6F) (169). The researchers are also working towards a lab-under-the-skin microneedle sensor arrays that can physically disrupt the outer layer of the skin in a minimally-invasive, painless manner (185). The interstitial fluid (ISF) microenvironment is closely related to blood, offering an attractive minimally-invasive skin compartment. A multiplexed microneedle sensor array platform has been developed for simultaneous transdermal monitoring of key health ISF biomarkers, such as glucose, alcohol, insulin, cortisol, lactate, and ketone (186). A self-powered glucose biosensor is already commercialized by Biolink company, which uses enzyme-modified carbon paste (CPE)-containing microneedle arrays with 9 hollow microneedles loaded with CPE (Figure 6N) (176). A microneedle-based sensor for Parkinson’s management is designed for continuous monitoring of L-dopa and precise dose regulation using different microneedles on the same patch (187). A minimally-invasive microneedle sensor was developed by integrating a microneedle patch with a 1.5 centimeter diameter for continuous monitoring of multiple chemical markers in subcutaneous tissue (Figure 6M) (175). In a very recent study, the reliability of the sweat-sensing system in noninvasively monitoring important biomarkers in the human body was confirmed by developing a wireless, battery-free, flexible, self-pumping sweat-sensing system for the long-term monitoring of changes in the status of levodopa and vitamin C in sweat, which can be useful in diagnosis, medication, and nutritional assessment (Figure 6A) (164). Three microneedle skin-offs companies, Biolink, Aquilx, and Nutramics, are focusing on drug monitoring in diseases like diabetes and Parkinson’s.

Meeting anatomically compliant power sources is crucial for progressing in wearable biomedical devices, which require miniaturization and flexibility to enable wearer activity. To have a complete control of the energy demand, scientists are developing flexible energy harvesting and storage systems, such as epidermal biofuel cells, tattoo-based batteries, supercapacitors, textile-based energy microgrids, and hybrid multi-modal energy systems (Figure 6D) (167, 188). Epidermal biofuel cells are highly stretchable and designed to scavenge bioenergy from wearers, like sweat. A team of researchers at UC San Diego developed a flexible, stretchable powerful “island-bridge” biofuel cell to increase power density (Figure 5E) (168). They also combined energy harvesting with energy storage on textiles, creating a hybrid energy system with biofuel cells on one side and supercapacitors on the other (Figure 6O) (177). High-performance flexible batteries are being developed, such as stretchable Zn-Ag_2_O batteries with a high reversible capacity density well suited for realizing chip-scale energy storage for integrated electronic systems. The ultimate goal is to combine complementary and synergistic energy harvester and storage modules to develop multi-modular wearable grid systems (189). Another example of a new self-sustainable wearable multi-modular bioenergy microgrid system that meet the demands of energy supply is a multi-module, textile-based energy-powering system with applications powered by complementary and synergistic energy harvesters and commensurate energy storage modules (Figure 6Q) (179). They recently combined all functionalities with an electrochromic display in the form of a unique patch design with high stretchability and changing color ability to visualize the sweat lactate concentration, which can be life-changing for cardiac care patients, organ transplant recipients, and people with diabetes. These non-invasive sensors could be used for fine-tuning electrolytes in athletes, peak performance, and military pilots in high-pressure jobs (190).

## Wearable/implantable biosensor applications in other organs

Researchers have tested a bioartificial kidney prototype, aiming to free kidney disease patients from dialysis machines and transplant waiting lists. The prototype, combining hemofilter and bioreactor components, was implanted for preclinical evaluation. The device powered by blood pressure without blood thinning or immunosuppressant drugs. The artificial kidney could offer complete mobility and better physiological outcomes than dialysis. The team plans to upscale the technology for more preclinical testing (191, 192).

Scientists have developed a multiplex COVID-19 diagnosis platform called SARS-CoV-2 RapidPlex, based on low-cost graphene-based laser-engraved technology. This system monitors virus antigen, antibodies, and inflammatory biomarkers in saliva and blood in less than 10 min. The system has good selectivity and accuracy compared to gold standard ELISA, distinguishing patients from healthy individuals and identifying influential biomarkers like CRP for monitoring COVID infection severity (193).

A study demonstrates that non-invasive magnets can effectively treat glioblastoma, a challenging brain cancer, with a 31% reduction in tumor volume in just over one month. The device, consisting of three rotating magnets called oncoscillators, is compact and simple for at-home treatment. The researchers created an oncomagnetic device, which covered the entire brain with minimal side effects. The tumor shrinks rapidly, with a 10% reduction after 3 days and a 30% reduction at 30 days. This is a significant step forward in cancer treatment, and it is hoped that treating cancer will soon be as easy as putting on a hat (194).

Envoy Medical’s Acclaim^®^ cochlear implant is an investigational device aimed at improving hearing for adults with moderate to profound sensorineural hearing loss. If approved by the FDA, it would be the first cochlear implant without external components, ensuring no loss or damage. The implant uses novel sensor technology from Envoy Medical’s Esteem osseointegrated active middle ear implant (AMEI), which was FDA-approved in 2010 (195).

Advanced endoscopes with imaging and therapy capabilities offer advantages but lack spatial resolution for diagnosing and treating small cancers. A multifunctional endoscope-based interventional system combines transparent bioelectronics with theranostic nanoparticles, enabling optical fluorescence-based mapping, electrical impedance and pH sensing, contact/temperature monitoring, radio frequency ablation, and localized photo/chemotherapy. This technology is useful for accurate detection, delineation, and targeted therapy of colon cancer, treating chronic inflammatory bowel diseases, and enhancing tumor detection accuracy (196).

Researchers have developed Pillsense, a swallowable device that detects gastrointestinal bleeding using a fluorescence detector. The world’s smallest floor imager, it uses an LED, lens, optical filter, and excitation filter. This innovative solution could prevent gastrointestinal bleeding in pre-symptomatic individuals without displaying symptoms (197).

There is a significant opportunity in the field of biometric sensors, which offer various modalities, form factors, and insights. Self-powered capabilities can be configured for some of these technologies, but further advancements are needed in sensing mechanisms. Examples include injectable biometric sensors for photoplethysmography, accelerometry, and thermometry (198), battery-free wireless ultrasonic sensors like endoleak sensing in aortic valves (199), and cardiac stem cells patched on the heart powered by ultrasound (200). Transdermal and breath biomarker sensing can detect body conditions like metabolic states or COVID-19 infection (201). Startups like NEXT system, ClearSense, Everactive, Dermisense, VitaFlo, Onda Vision, and Olftech are actively pursuing technology development. Future opportunities include new energy harvesting modes, non-invasive sensing modalities like heat flux sensors, blood pressure sensors, artificial intelligence, and machine learning. Additionally, reconfigurable sensor systems can be used to change the sampling rate or sensing target based on context.

## Conclusion

While the integration of precision medicine and wearable biosensors holds immense promise, several challenges must be addressed. These include issues related to data privacy, the need for standardized data formats, and the importance of educating both healthcare providers and the general public about the potential benefits and limitations of these technologies. The integration of biosensors into everyday life presents challenges, including the need for wearable devices to be comfortable, unobtrusive, and fashionably acceptable, and implantables to navigate biocompatibility and long-term stability. Accuracy and reliability of biosensor data are crucial for clinical utility, and bridging gaps between medical, engineering, and data science disciplines is essential. Ethical concerns regarding privacy, consent, and data ownership are crucial for the widespread acceptance and ethical deployment of biosensors in precision medicine.

Future biosensors may leverage advancements in sensing technologies, such as nanoscale sensors and innovative biomaterials, to enhance the specificity and sensitivity of biosensors. Integrated health platforms that combine data from wearable and implantable biosensors with electronic health records could provide healthcare professionals with a comprehensive view of a patient’s health, facilitating more informed and personalized treatment strategies.

Predictive analytics and artificial intelligence are key to extracting meaningful insights from biosensor data, with machine learning algorithms aiding in identifying patterns, predicting disease trajectories, and optimizing treatment plans. As technology evolves, there is a growing potential to empower individuals to actively participate in their healthcare, fostering health literacy and self-management.

In conclusion, while challenges persist, the future prospects of wearable and implantable biosensors in precision medicine are promising. Addressing current obstacles through collaborative efforts and embracing technological advancements could pave the way for a healthcare landscape where personalized interventions based on real-time, accurate data become the standard rather than the exception.

## Author contributions

EG: Writing – original draft. ZN: Writing – review & editing. H-PD: Conceptualization, Writing – review & editing. HR: Validation, Visualization, Investigation, Writing – review & editing. ZA: Resources, Conceptualization, Writing – review & editing.

## References

[1] Naithani N Sinha S Misra P Vasudevan B Sahu R. Precision medicine: concept and tools. Med J Armed Forces India. (2021) 77:249–57. doi: 10.1016/j.mjafi.2021.06.021, PMID: 34305276 PMC8282508

[2] Mirnezami R Wafi A. Chapter 54—the future of precision medicine In: Faintuch J Faintuch S, editors. Precision medicine for investigators, practitioners and providers. India: Academic Press. (2020) 561–9.

[3] Sharma A Singh A Gupta V Arya S. Advancements and future prospects of wearable sensing technology for healthcare applications. Sens Diagn. (2022) 1:387–404. doi: 10.1039/D2SD00005A

[4] Xu S Kim J Walter JR Ghaffari R Rogers JA. Translational gaps and opportunities for medical wearables in digital health. Sci Transl Med. (2022) 14:eabn6036. doi: 10.1126/scitranslmed.abn6036 36223451 PMC10193448

[5] Gray M Meehan J Ward C Langdon SP Kunkler IH Murray A. Implantable biosensors and their contribution to the future of precision medicine. Vet J. (2018) 239:21–9. doi: 10.1016/j.tvjl.2018.07.011 30197105

[6] Yogev D Goldberg T Arami A Tejman-Yarden S Winkler TE Maoz BM. Current state of the art and future directions for implantable sensors in medical technology: clinical needs and engineering challenges. APL Bioeng. (2023) 7:031506. doi: 10.1063/5.0152290, PMID: 37781727 PMC10539032

[7] Farina L. Systems precision medicine: putting the pieces back together. Systems. (2023) 11:367. doi: 10.3390/systems11070367

[8] Tesi B Boileau C Boycott KM Canaud G Caulfield M Choukair D. Precision medicine in rare diseases: what is next? J Intern Med. (2023) 294:397–412. doi: 10.1111/joim.13655 37211972

[9] Liu Y Li J Xiao S Liu Y Bai M Gong L. Revolutionizing precision medicine: exploring wearable sensors for therapeutic drug monitoring and personalized therapy. Biosensors. (2023) 13:726. doi: 10.3390/bios13070726, PMID: 37504123 PMC10377150

[10] Tyler J Choi SW Tewari M. Real-time, personalized medicine through wearable sensors and dynamic predictive modeling: a new paradigm for clinical medicine. Curr Opin Syst Biol. (2020) 20:17–25. doi: 10.1016/j.coisb.2020.07.001, PMID: 32984661 PMC7515448

[11] Seyhan AA Carini C. Are innovation and new technologies in precision medicine paving a new era in patients centric care? J Transl Med. (2019) 17:114. doi: 10.1186/s12967-019-1864-9, PMID: 30953518 PMC6451233

[12] Pires IM Dobre C Zdravevski E Garcia NM. Editorial: wearable and mobile data analysis methodologies for personalized medicine. Front Digit Health. (2023) 5:1271659. doi: 10.3389/fdgth.2023.1271659 37800089 PMC10548372

[13] Haleem A Javaid M Singh RP Suman R Rab S. Biosensors applications in medical field: a brief review. Sens Int. (2021) 2:100100. doi: 10.1016/j.sintl.2021.100100

[14] Giansanti D. Precision medicine 2.0: how digital health and AI are changing the game. J Pers Med. (2023) 13:1057. doi: 10.3390/jpm13071057 37511670 PMC10381472

[15] Wang W-H Hsu W-S. Integrating artificial intelligence and wearable IoT system in long-term care environments. Sensors. (2023) 23:5913. doi: 10.3390/s23135913, PMID: 37447763 PMC10346723

[16] Smith AA Li R Tse ZTH. Reshaping healthcare with wearable biosensors. Sci Rep. (2023) 13:4998. doi: 10.1038/s41598-022-26951-z, PMID: 36973262 PMC10043012

[17] Gomes N Pato M Lourenço AR Datia N. A survey on wearable sensors for mental health monitoring. Sensors. (2023) 23:1330. doi: 10.3390/s23031330, PMID: 36772370 PMC9919280

[18] Prystowsky EN Korzinov L Baumann E Denis S Jaime ME James J. System and method for processing and presenting arrhythmia information to facilitate heart arrhythmia identification and treatment In: Google Patents. porio jan. T56489769. US patent. (2007)

[19] Grouios G Ziagkas E Loukovitis A Chatzinikolaou K Koidou E. Accelerometers in our pocket: does smartphone accelerometer technology provide accurate data? Sensors. (2022) 23:192. doi: 10.3390/s23010192, PMID: 36616798 PMC9824767

[20] Da He D Winokur ES Sodini CG. (2012). An ear-worn continuous ballistocardiogram (BCG) sensor for cardiovascular monitoring. 2012 Annual International Conference of the IEEE Engineering in Medicine and Biology Society IEEE. 10.1109/EMBC.2012.6347123 PMC4384813 23367058

[21] Carek AM Conant J Joshi A Kang H Inan OT. SeismoWatch: wearable cuffless blood pressure monitoring using pulse transit time. Proc ACM Interact Mob Wearable Ubiquitous Technol. (2017) 1:1–16. doi: 10.1145/3130905, PMID: 30556049 PMC6292433

[22] Etemadi M Inan OT. Wearable ballistocardiogram and seismocardiogram systems for health and performance. J Appl Physiol. (2018) 124:452–61. doi: 10.1152/japplphysiol.00298.2017, PMID: 28798198 PMC5867366

[23] Budidha K Kyriacou P. The human ear canal: investigation of its suitability for monitoring photoplethysmographs and arterial oxygen saturation. Physiol Meas. (2014) 35:111–28. doi: 10.1088/0967-3334/35/2/111, PMID: 24399082

[24] Castaneda D Esparza A Ghamari M Soltanpur C Nazeran H. A review on wearable photoplethysmography sensors and their potential future applications in health care. Int J Biosens Bioelectron. (2018) 4:195–202. doi: 10.15406/ijbsbe.2018.04.00125, PMID: 30906922 PMC6426305

[25] Proesmans T Mortelmans C Van Haelst R Verbrugge F Vandervoort P Vaes B. Mobile phone-based use of the photoplethysmography technique to detect atrial fibrillation in primary care: diagnostic accuracy study of the FibriCheck app. JMIR Mhealth Uhealth. (2019) 7:e12284. doi: 10.2196/12284, PMID: 30916656 PMC6456825

[26] Hamad AKS. New technologies for detection and management of atrial fibrillation. J Saudi Heart Assoc. (2021) 33:169–76. doi: 10.37616/2212-5043.1256, PMID: 34249609 PMC8260036

[27] Ding EY Han D Whitcomb C Bashar SK Adaramola O Soni A. Accuracy and usability of a novel algorithm for detection of irregular pulse using a smartwatch among older adults: observational study. JMIR Cardio. (2019) 3:e13850. doi: 10.2196/13850, PMID: 31758787 PMC6834225

[28] Jacobsen M Dembek TA Ziakos A-P Gholamipoor R Kobbe G Kollmann M. Reliable detection of atrial fibrillation with a medical wearable during inpatient conditions. Sensors. (2020) 20:5517. doi: 10.3390/s20195517, PMID: 32993132 PMC7583973

[29] Lee SP Ha G Wright DE Ma Y Sen-Gupta E Haubrich NR. Highly flexible, wearable, and disposable cardiac biosensors for remote and ambulatory monitoring. npj Digit Med. (2018) 1:2. doi: 10.1038/s41746-017-0009-x 31304288 PMC6550217

[30] Kim CS Yang HM Lee J Lee GS Choi H Kim YJ. Self-powered wearable electrocardiography using a wearable thermoelectric power generator. ACS Energy Lett. (2018) 3:501–7. doi: 10.1021/acsenergylett.7b01237

[31] Abdou A Krishnan S. Horizons in single-lead ECG analysis from devices to data. Fronti Signal Process. (2022) 2:866047. doi: 10.3389/frsip.2022.866047

[32] Yiu C Liu Y Zhang C Zhou J Jia H Wong TH. Soft, stretchable, wireless intelligent three-lead electrocardiograph monitors with feedback functions for warning of potential heart attack. SmartMat. (2022) 3:668–84. doi: 10.1002/smm2.1114

[33] Dilaveris P Tsioufis C. The single-lead 14-day ECG patch EZYPRO^®^: a new kid in the block. Int J Cardiol. (2021) 332:89–90. doi: 10.1016/j.ijcard.2021.03.069 33798628

[34] Hansen IH Hoppe K Gjerde A Kanters JK Sorensen HB. (2015). Comparing twelve-lead electrocardiography with close-to-heart patch based electrocardiography. 2015 37th annual international conference of the IEEE engineering in medicine and biology society (EMBC): IEEE. 10.1109/EMBC.2015.7318366 26736266

[35] Chua S-K Chen L-C Lien L-M Lo H-M Liao Z-Y Chao S-P. Comparison of arrhythmia detection by 24-hour Holter and 14-day continuous electrocardiography patch monitoring. Acta Cardiol Sin. (2020) 36:251–9. doi: 10.6515/ACS.202005_36(3).20190903A, PMID: 32425440 PMC7220965

[36] Boszko M Osak G Żurawska N Skoczylas K Krzowski B Wróblewski G. Assessment of a new KoMaWo electrode-patch configuration accuracy and review of the literature. J Electrocardiol. (2022) 75:82–7. doi: 10.1016/j.jelectrocard.2022.07.004, PMID: 35918203

[37] Garabelli P Stavrakis S Po S. Smartphone-based arrhythmia monitoring. Curr Opin Cardiol. (2017) 32:53–7. doi: 10.1097/HCO.0000000000000350 27875477

[38] Chan PH Wong CK Poh YC Pun L Leung WWC Wong YF. Diagnostic performance of a smartphone-based photoplethysmographic application for atrial fibrillation screening in a primary care setting. J Am Heart Assoc. (2016) 5:e003428. doi: 10.1161/JAHA.116.003428, PMID: 27444506 PMC5015379

[39] Sana F Isselbacher EM Singh JP Heist EK Pathik B Armoundas AA. Wearable devices for ambulatory cardiac monitoring: JACC state-of-the-art review. J Am Coll Cardiol. (2020) 75:1582–92. doi: 10.1016/j.jacc.2020.01.046, PMID: 32241375 PMC7316129

[40] Passler S Müller N Senner V. In-ear pulse rate measurement: a valid alternative to heart rate derived from electrocardiography? Sensors. (2019) 19:3641. doi: 10.3390/s19173641, PMID: 31438600 PMC6749408

[41] Hodkinson A Kontopantelis E Adeniji C Van Marwijk H McMillian B Bower P. Interventions using wearable physical activity trackers among adults with cardiometabolic conditions: a systematic review and meta-analysis. JAMA Netw Open. (2021) 4:e2116382. doi: 10.1001/jamanetworkopen.2021.16382 34283229 PMC9387744

[42] Choi J-H Kim S-W Seo J Sun Y Jung W-S Park H-Y. Effects of a mobile-health exercise intervention on body composition, vascular function, and autonomic nervous system function in obese women: a randomized controlled trial. J Multidiscip Healthc. (2023) 16:1601–15. doi: 10.2147/JMDH.S406905 37313274 PMC10259526

[43] Vaes B Stalpaert S Tavernier K Thaels B Lapeire D Mullens W. The diagnostic accuracy of the MyDiagnostick to detect atrial fibrillation in primary care. BMC Fam Pract. (2014) 15:1–7. doi: 10.1186/1471-2296-15-113 24913608 PMC4069340

[44] Svennberg E Stridh M Engdahl J Al-Khalili F Friberg L Frykman V. Safe automatic one-lead electrocardiogram analysis in screening for atrial fibrillation. Europace. (2017) 19:1449–53. doi: 10.1093/europace/euw286 28339578

[45] Turakhia MP Desai M Hedlin H Rajmane A Talati N Ferris T. Rationale and design of a large-scale, app-based study to identify cardiac arrhythmias using a smartwatch: The Apple Heart Study. Am Heart J. (2019) 207:66–75. doi: 10.1016/j.ahj.2018.09.002, PMID: 30392584 PMC8099048

[46] Provencio A Gil MÁC. Smartwatch electrocardiogram records ST depression, premature ventricular complexes, and ventricular fibrillation. Lancet. (2022) 400:e12. doi: 10.1016/S0140-6736(22)01978-X, PMID: 36366887

[47] Muhlestein JB Anderson JL Bethea CF Severance HW Mentz RJ Barsness GW. Feasibility of combining serial smartphone single-lead electrocardiograms for the diagnosis of ST-elevation myocardial infarction. Am Heart J. (2020) 221:125–35. doi: 10.1016/j.ahj.2019.12.016, PMID: 31986289

[48] Amir O Ben-Gal T Weinstein JM Schliamser J Burkhoff D Abbo A. Evaluation of remote dielectric sensing (ReDS) technology-guided therapy for decreasing heart failure re-hospitalizations. Int J Cardiol. (2017) 240:279–84. doi: 10.1016/j.ijcard.2017.02.120, PMID: 28341372

[49] Hauguel-Moreau M Naudin C N’Guyen L Squara P Rosencher J Makowski S. Smart bracelet to assess physical activity after cardiac surgery: a prospective study. PLoS One. (2020) 15:e0241368. doi: 10.1371/journal.pone.0241368, PMID: 33259484 PMC7707519

[50] Heizmann A-N Chapelle C Laporte S Roche F Hupin D Le Hello C. Impact of wearable device-based interventions with feedback for increasing daily walking activity and physical capacities in cardiovascular patients: a systematic review and meta-analysis of randomised controlled trials. BMJ Open. (2023) 13:e069966. doi: 10.1136/bmjopen-2022-069966, PMID: 37433730 PMC10347518

[51] Widmer RJ Collins NM Collins CS West CP Lerman LO Lerman A. Digital health interventions for the prevention of cardiovascular disease: A systematic review and meta-analysis. Mayo Clin Proc. (2015) 90:469–80. doi: 10.1016/j.mayocp.2014.12.026 25841251 PMC4551455

[52] Sattar Y Zghouzi M Suleiman A-RM Sheikh A Kupferman J Sarfraz A. Efficacy of remote dielectric sensing (ReDS) in the prevention of heart failure rehospitalizations: a meta-analysis. J Community Hosp Intern Med Perspect. (2021) 11:646–52. doi: 10.1080/20009666.2021.1955451 34567456 PMC8462919

[53] Johnson KT Picard RW. Advancing neuroscience through wearable devices. Neuron. (2020) 108:8–12. doi: 10.1016/j.neuron.2020.09.030 33058768

[54] Mestais CS Charvet G Sauter-Starace F Foerster M Ratel D Benabid AL. WIMAGINE: wireless 64-channel ECoG recording implant for long term clinical applications. IEEE Trans Neural Syst Rehabil Eng. (2014) 23:10–21. doi: 10.1109/TNSRE.2014.2333541, PMID: 25014960

[55] Luo J Xue N Chen J. A review: research progress of neural probes for brain research and brain-computer Interface. Biosensors. (2022) 12:1167. doi: 10.3390/bios12121167, PMID: 36551135 PMC9775442

[56] Drew L. The brain-reading devices helping paralysed people to move, talk and touch. Nature. (2022) 604:416–9. doi: 10.1038/d41586-022-01047-w, PMID: 35444327

[57] Maiseli B Abdalla AT Massawe LV Mbise M Mkocha K Nassor NA. Brain-computer interface: trend, challenges, and threats. Brain Inform. (2023) 10:20. doi: 10.1186/s40708-023-00199-3, PMID: 37540385 PMC10403483

[58] Woeppel K Hughes C Herrera AJ Eles JR Tyler-Kabara EC Gaunt RA. Explant analysis of Utah electrode arrays implanted in human cortex for brain-computer-interfaces. Front Bioeng Biotechnol. (2021) 9:759711. doi: 10.3389/fbioe.2021.759711 34950640 PMC8688945

[59] Simeral JD Hosman T Saab J Flesher SN Vilela M Franco B. Home use of a percutaneous wireless intracortical brain-computer interface by individuals with tetraplegia. IEEE Trans Biomed Eng. (2021) 68:2313–25. doi: 10.1109/TBME.2021.3069119, PMID: 33784612 PMC8218873

[60] Zhang J Liu X Xu W Luo W Li M Chu F. Stretchable transparent electrode arrays for simultaneous electrical and optical interrogation of neural circuits *in vivo*. Nano Lett. (2018) 18:2903–11. doi: 10.1021/acs.nanolett.8b00087, PMID: 29608857

[61] Kim Y Alimperti S Choi P Noh M. An inkjet printed flexible wlectrocorticography (ECoG) microelectrode array on a thin parylene-C. Sensors. (2022) 22:1277. doi: 10.3390/s22031277 35162023 PMC8838719

[62] Lee WR Im C Park HY Seo JM Kim JM. Fabrication of convex PDMS-parylene microstructures for conformal contact of planar micro-electrode array. Polymers. (2019) 11:1436. doi: 10.3390/polym11091436, PMID: 31480664 PMC6780241

[63] Diaz-Botia CA Luna LE Neely RM Chamanzar M Carraro C Carmena JM. A silicon carbide array for electrocorticography and peripheral nerve recording. J Neural Eng. (2017) 14:056006. doi: 10.1088/1741-2552/aa7698, PMID: 28573982

[64] Zhao Z Zhu H Li X Sun L He F Chung JE. Ultraflexible electrode arrays for months-long high-density electrophysiological mapping of thousands of neurons in rodents. Nat Biomed Eng. (2023) 7:520–32. doi: 10.1038/s41551-022-00941-y, PMID: 36192597 PMC10067539

[65] Tacca N Nassour J Ehrlich SK Berberich N Cheng G. Neuro-cognitive assessment of intentional control methods for a soft elbow exosuit using error-related potentials. J Neuroeng Rehabil. (2022) 19:124. doi: 10.1186/s12984-022-01098-0, PMID: 36369025 PMC9652996

[66] Seo D Neely RM Shen K Singhal U Alon E Rabaey JM. Wireless recording in the peripheral nervous system with ultrasonic neural dust. Neuron. (2016) 91:529–39. doi: 10.1016/j.neuron.2016.06.034, PMID: 27497221

[67] Young MJ Lin DJ Hochberg LR. Brain-computer interfaces in neurorecovery and neurorehabilitation. Semin Neurol. (2021) 41:206–16. doi: 10.1055/s-0041-1725137 33742433 PMC8768507

[68] Kalia SK Sankar T Lozano AM. Deep brain stimulation for Parkinson's disease and other movement disorders. Curr Opin Neurol. (2013) 26:374–80. doi: 10.1097/WCO.0b013e3283632d08 23817213

[69] Bergeron D Iorio-Morin C Bonizzato M Lajoie G Orr Gaucher N Racine É. Use of invasive brain-computer interfaces in pediatric neurosurgery: technical and ethical considerations. J Child Neurol. (2023) 38:223–38. doi: 10.1177/08830738231167736, PMID: 37116888 PMC10226009

[70] Rapeaux AB Constandinou TG. Implantable brain machine interfaces: first-in-human studies, technology challenges and trends. Curr Opin Biotechnol. (2021) 72:102–11. doi: 10.1016/j.copbio.2021.10.001, PMID: 34749248

[71] Dubey A Ray S. Cortical electrocorticogram (ECoG) is a local signal. J Neurosci. (2019) 39:4299–311. doi: 10.1523/JNEUROSCI.2917-18.2019, PMID: 30914446 PMC6538865

[72] Shahabi H Nair DR Leahy RM. Multilayer brain networks can identify the epileptogenic zone and seizure dynamics. eLife. (2023) 12:e68531. doi: 10.7554/eLife.68531, PMID: 36929752 PMC10065796

[73] Mercier MR Dubarry A-S Tadel F Avanzini P Axmacher N Cellier D. Advances in human intracranial electroencephalography research, guidelines and good practices. NeuroImage. (2022) 260:119438. doi: 10.1016/j.neuroimage.2022.119438 35792291 PMC10190110

[74] Yanagisawa T Hirata M Saitoh Y Kishima H Matsushita K Goto T. Electrocorticographic control of a prosthetic arm in paralyzed patients. Ann Neurol. (2012) 71:353–61. doi: 10.1002/ana.22613, PMID: 22052728

[75] Liu K Yu Y Liu Y Tang J Liang X Chu X. A novel brain-controlled wheelchair combined with computer vision and augmented reality. Biomed Eng Online. (2022) 21:1–20. doi: 10.1186/s12938-022-01020-8 35883092 PMC9327337

[76] Melcer EF Astolfi MT Remaley M Berenzweig A Giurgica-Tiron T. (2018). CTRL-labs: hand activity estimation and real-time control from neuromuscular signals. Extended Abstracts of the 2018 CHI Conference on Human Factors in Computing Systems.

[77] Narayana S Prasad RV Warmerdam K. Mind your thoughts: BCI using single EEG electrode. IET Cyber-Phys Syst: Theory Appl. (2019) 4:164–72. doi: 10.1049/iet-cps.2018.5059

[78] Dhiman R. Machine learning techniques for electroencephalogram based brain-computer interface: a systematic literature review. Meas: Sens. (2023) 28:100823. doi: 10.1016/j.measen.2023.100823

[79] Lorach H Galvez A Spagnolo V Martel F Karakas S Intering N. Walking naturally after spinal cord injury using a brain-spine interface. Nature. (2023) 618:126–33. doi: 10.1038/s41586-023-06094-5, PMID: 37225984 PMC10232367

[80] Mahmood M Mzurikwao D Kim Y-S Lee Y Mishra S Herbert R. Fully portable and wireless universal brain-machine interfaces enabled by flexible scalp electronics and deep learning algorithm. Nat Mach Intell. (2019) 1:412–22. doi: 10.1038/s42256-019-0091-7

[81] Pais-Vieira M Lebedev M Kunicki C Wang J Nicolelis MA. A brain-to-brain interface for real-time sharing of sensorimotor information. Sci Rep. (2013) 3:1319. doi: 10.1038/srep01319, PMID: 23448946 PMC3584574

[82] Ramakrishnan A Ifft PJ Pais-Vieira M Byun YW Zhuang KZ Lebedev MA. Computing arm movements with a monkey brainet. Sci Rep. (2015) 5:10767. doi: 10.1038/srep10767, PMID: 26158523 PMC4497496

[83] Brandmeyer T Delorme A. Meditation and neurofeedback. Front Psychol. (2013) 4:688:688. doi: 10.3389/fpsyg.2013.00688 24109463 PMC3791377

[84] Nijholt A. Introduction: brain-computer interfaces for artistic expression In: Brain art. Cham: Springer (2019). 1–29.

[85] Huang X Yin E Wang Y Saab R Gao X. (2017). A mobile EEG system for practical applications. 2017 IEEE Global Conference on Signal and Information Processing (GlobalSIP): IEEE.

[86] Song S Nordin AD. Mobile electroencephalography for studying neural control of human locomotion. Front Hum Neurosci. (2021) 15:749017. doi: 10.3389/fnhum.2021.749017, PMID: 34858154 PMC8631362

[87] Moses DA Metzger SL Liu JR Anumanchipalli GK Makin JG Sun PF. Neuroprosthesis for decoding speech in a paralyzed person with anarthria. N Engl J Med. (2021) 385:217–27. doi: 10.1056/NEJMoa2027540, PMID: 34260835 PMC8972947

[88] Tolosa E Garrido A Scholz SW Poewe W. Challenges in the diagnosis of Parkinson’s disease. Lancet Neurol. (2021) 20:385–97. doi: 10.1016/S1474-4422(21)00030-2, PMID: 33894193 PMC8185633

[89] Sigcha L Borzì L Amato F Rechichi I Ramos-Romero C Cárdenas A. Deep learning and wearable sensors for the diagnosis and monitoring of Parkinson’s disease: a systematic review. Expert Syst Appl. (2023) 229:120541. doi: 10.1016/j.eswa.2023.120541

[90] Cohen S Bataille LR Martig AK. Enabling breakthroughs in Parkinson’s disease with wearable technologies and big data analytics. Mhealth. (2016) 2:2. doi: 10.21037/mhealth.2016.04.02 28293596 PMC5344108

[91] Pennati GV Bergling H Carment L Borg J Lindberg PG Palmcrantz S. Effects of 60 min electrostimulation with the EXOPULSE mollii suit on objective signs of spasticity. Front Neurol. (2021) 12:706610. doi: 10.3389/fneur.2021.706610 34721255 PMC8554021

[92] Pham MT Campbell TA Dorfman N Torgerson L Kostick-Quenet K Blumenthal-Barby J. Clinician perspectives on levels of evidence and oversight for deep brain stimulation for treatment-resistant childhood OCD. J Obsessive Compuls Relat Disord. (2023) 39:100830. doi: 10.1016/j.jocrd.2023.100830, PMID: 37781644 PMC10538479

[93] Tan XS Pierres F Dallman-Porter A Hardie-Brown W Kwon K-Y. Focused vibrotactile stimulation with cueing effect on freezing of gait in Parkinson’s disease: two case reports. J Mov Disord. (2021) 14:236–8. doi: 10.14802/jmd.21076, PMID: 34488302 PMC8490196

[94] Chung JE Joo HR Fan JL Liu DF Barnett AH Chen S. High-density, long-lasting, and multi-region electrophysiological recordings using polymer electrode arrays. Neuron. (2019) 101:21–31.e5. e5. doi: 10.1016/j.neuron.2018.11.002, PMID: 30502044 PMC6326834

[95] Kleen JK Chung JE Sellers KK Zhou J Triplett M Lee K. Bidirectional propagation of low frequency oscillations over the human hippocampal surface. Nat Commun. (2021) 12:2764. doi: 10.1038/s41467-021-22850-5, PMID: 33980852 PMC8115072

[96] Muller L Felix S Shah KG Lee K Pannu S Chang EF. (2016). Thin-film, high-density micro-electrocorticographic decoding of a human cortical gyrus. 2016 38th Annual International Conference of the IEEE Engineering in Medicine and Biology Society (EMBC): IEEE. 10.1109/EMBC.2016.7591001 PMC5789448 28268617

[97] Metzger SL Liu JR Moses DA Dougherty ME Seaton MP Littlejohn KT. Generalizable spelling using a speech neuroprosthesis in an individual with severe limb and vocal paralysis. Nat Commun. (2022) 13:6510. doi: 10.1038/s41467-022-33611-3, PMID: 36347863 PMC9643551

[98] Neely RM Piech DK Santacruz SR Maharbiz MM Carmena JM. Recent advances in neural dust: towards a neural interface platform. Curr Opin Neurobiol. (2018) 50:64–71. doi: 10.1016/j.conb.2017.12.010, PMID: 29331738

[99] Facciorusso S Spina S Reebye R Turolla A Calabrò RS Fiore P. Sensor-based rehabilitation in neurological diseases: a bibliometric analysis of research trends. Brain Sci. (2023) 13:724. doi: 10.3390/brainsci13050724, PMID: 37239196 PMC10216556

[100] Shin H Seo H Chung WG Joo BJ Jang J Park J-U. Recent progress on wearable point-of-care devices for ocular systems. Lab Chip. (2021) 21:1269–86. doi: 10.1039/D0LC01317J, PMID: 33704299

[101] Mirzajani H Mirlou F Istif E Singh R Beker L. Powering smart contact lenses for continuous health monitoring: recent advancements and future challenges. Biosens Bioelectron. (2022) 197:113761. doi: 10.1016/j.bios.2021.113761, PMID: 34800926

[102] Tseng RC Chen C-C Hsu S-M Chuang H-S. Contact-lens biosensors. Sensors. (2018) 18:2651. doi: 10.3390/s18082651 30104496 PMC6111605

[103] Lin B Wang M Zhao C Wang S Chen K Li X. Flexible organic integrated electronics for self-powered multiplexed ocular monitoring. npj Flex Electron. (2022) 6:77. doi: 10.1038/s41528-022-00211-6

[104] Yetisen AK Jiang N Castaneda Gonzalez CM Erenoglu ZI Dong J Dong X. Scleral lens sensor for ocular electrolyte analysis. Adv Mater. (2020) 32:e1906762. doi: 10.1002/adma.201906762, PMID: 31834667

[105] Chu M Shirai T Takahashi D Arakawa T Kudo H Sano K. Biomedical soft contact-lens sensor for in situ ocular biomonitoring of tear contents. Biomed Microdevices. (2011) 13:603–11. doi: 10.1007/s10544-011-9530-x, PMID: 21475940

[106] Ku M Kim J Won J-E Kang W Park Y-G Park J. Smart, soft contact lens for wireless immunosensing of cortisol. Sci Adv. (2020) 6:eabb2891. doi: 10.1126/sciadv.abb2891 32923592 PMC7455488

[107] Li S Zhu Y Haghniaz R Kawakita S Guan S Chen J. A microchambers containing contact lens for the noninvasive detection of tear exosomes. Adv Funct Mater. (2022) 32:2206620. doi: 10.1002/adfm.202206620

[108] Moreddu R Elsherif M Adams H Moschou D Cordeiro MF Wolffsohn JS. Integration of paper microfluidic sensors into contact lenses for tear fluid analysis. Lab Chip. (2020) 20:3970–9. doi: 10.1039/D0LC00438C, PMID: 32944726

[109] Kim J Kim J Ku M Cha E Ju S Park WY. Intraocular pressure monitoring following islet transplantation to the anterior chamber of the eye. Nano Lett. (2019) 20:1517–25. doi: 10.1021/acs.nanolett.9b03605, PMID: 31750664

[110] Kim TY Mok JW Hong SH Jeong SH Choi H Shin S. Wireless theranostic smart contact lens for monitoring and control of intraocular pressure in glaucoma. Nat Commun. (2022) 13:6801. doi: 10.1038/s41467-022-34597-8 36357417 PMC9649789

[111] Mun J Kim TY Myung D Hahn SK. Smart contact lens containing hyaluronate–rose bengal conjugate for biophotonic myopia vision correction. Biomater Sci. (2022) 10:4997–5005. doi: 10.1039/D2BM00584K, PMID: 35815427 PMC9707406

[112] Kim J Park J Park Y-G Cha E Ku M An HS. A soft and transparent contact lens for the wireless quantitative monitoring of intraocular pressure. Nat Biomed Eng. (2021) 5:772–82. doi: 10.1038/s41551-021-00719-8, PMID: 33941897

[113] Lee GH Jeon C Mok JW Shin S Kim SK Han HH. Smart wireless near-infrared light emitting contact lens for the treatment of diabetic retinopathy. Adv Sci. (2022) 9:e2103254. doi: 10.1002/advs.202103254, PMID: 35092362 PMC8948592

[114] Xie M Yao G Zhang T Wang Q Mo X Dong Q. Multifunctional flexible contact lens for eye health monitoring using inorganic magnetic oxide nanosheets. J Nanobiotechnol. (2022) 20:202. doi: 10.1186/s12951-022-01415-8, PMID: 35477463 PMC9044588

[115] Moreddu R Wolffsohn JS Vigolo D Yetisen AK. Laser-inscribed contact lens sensors for the detection of analytes in the tear fluid. Sensors Actuators B. (2020) 317:128183. doi: 10.1016/j.snb.2020.128183

[116] Liu Z. Contact lenses having two-electrode electrochemical sensors In: Google Patents. Adrio. A23547868. US patent. (2014)

[117] Donora M Quintero AV De Smet H Underwood I. Spatiotemporal electrochemical sensing in a smart contact lens. Sensors Actuators B. (2020) 303:127203. doi: 10.1016/j.snb.2019.127203

[118] Raveendran R Prabakaran L Senthil R Yesudhason BV Dharmalingam S Sathyaraj WV. Current innovations in intraocular pressure monitoring biosensors for diagnosis and treatment of glaucoma—novel strategies and future perspectives. Biosensors. (2023) 13:663. doi: 10.3390/bios13060663, PMID: 37367028 PMC10296311

[119] An H Chen L Liu X Wang X Liu Y Wu Z. High-sensitivity liquid-metal-based contact lens sensor for continuous intraocular pressure monitoring. J Micromech Microeng. (2021) 31:035006. doi: 10.1088/1361-6439/abd8e0

[120] An H Chen L Liu X Zhao B Zhang H Wu Z. Microfluidic contact lenses for unpowered, continuous and non-invasive intraocular pressure monitoring. Sensors Actuators A. (2019) 295:177–87. doi: 10.1016/j.sna.2019.04.050

[121] Zhang J Kim K Kim HJ Meyer D Park W Lee SA. Smart soft contact lenses for continuous 24-hour monitoring of intraocular pressure in glaucoma care. Nat Commun. (2022) 13:5518. doi: 10.1038/s41467-022-33254-4, PMID: 36127347 PMC9489713

[122] Elsherif M Moreddu R Alam F Salih AE Ahmed I Butt H. Wearable smart contact lenses for continual glucose monitoring: a review. Front Med. (2022) 9:858784. doi: 10.3389/fmed.2022.858784, PMID: 35445050 PMC9013844

[123] Wen X Liu Q Zhang M. Innovative advancement of contact lenses for noninvasive diagnosis and therapy: a mini review. MedComm–Biomater Appl. (2023) 2:e40. doi: 10.1002/mba2.40

[124] Kim S Jeon H-J Park S Lee DY Chung E. Tear glucose measurement by reflectance spectrum of a nanoparticle embedded contact lens. Sci Rep. (2020) 10:8254. doi: 10.1038/s41598-020-65103-z, PMID: 32427894 PMC7237479

[125] Yang X Yao H Zhao G Ameer GA Sun W Yang J. Flexible, wearable microfluidic contact lens with capillary networks for tear diagnostics. J Mater Sci. (2020) 55:9551–61. doi: 10.1007/s10853-020-04688-2

[126] Roostaei N Hamidi S. Two-dimensional biocompatible plasmonic contact lenses for color blindness correction. Sci Rep. (2022) 12:2037. doi: 10.1038/s41598-022-06089-8, PMID: 35132172 PMC8821612

[127] Jang J Kim J Shin H Park Y-G Joo BJ Seo H. Smart contact lens and transparent heat patch for remote monitoring and therapy of chronic ocular surface inflammation using mobiles. Sci Adv. (2021) 7:eabf7194. doi: 10.1126/sciadv.abf7194, PMID: 33789904 PMC8011975

[128] Ishihara K Shi X Fukazawa K Yamaoka T Yao G Wu JY. Biomimetic-engineered silicone hydrogel contact lens materials. ACS Appl Bio Mater. (2023) 6:3600–16. doi: 10.1021/acsabm.3c00296, PMID: 37616500 PMC10521029

[129] Dennyson Savariraj A Salih A Alam F Elsherif M AlQattan B Khan AA. Ophthalmic sensors and drug delivery. ACS Sens. (2021) 6:2046–76. doi: 10.1021/acssensors.1c00370, PMID: 34043907 PMC8294612

[130] Yoon H Xuan X Jeong S Park JY. Wearable, robust, non-enzymatic continuous glucose monitoring system and its *in vivo* investigation. Biosens Bioelectron. (2018) 117:267–75. doi: 10.1016/j.bios.2018.06.008, PMID: 29909198

[131] Yang J Gong X Chen S Zheng Y Peng L Liu B. Development of smartphone-controlled and microneedle-based wearable continuous glucose monitoring system for home-care diabetes management. ACS Sens. (2023) 8:1241–51. doi: 10.1021/acssensors.2c02635, PMID: 36821704

[132] Chu MX Miyajima K Takahashi D Arakawa T Sano K Sawada S. Soft contact lens biosensor for in situ monitoring of tear glucose as non-invasive blood sugar assessment. Talanta. (2011) 83:960–5. doi: 10.1016/j.talanta.2010.10.055, PMID: 21147344

[133] Kownacka AE Vegelyte D Joosse M Anton N Toebes BJ Lauko J. Clinical evidence for use of a noninvasive biosensor for tear glucose as an alternative to painful finger-prick for diabetes management utilizing a biopolymer coating. Biomacromolecules. (2018) 19:4504–11. doi: 10.1021/acs.biomac.8b01429, PMID: 30350599 PMC6234487

[134] Arakawa T Kuroki Y Nitta H Chouhan P Toma K Sawada S. Mouthguard biosensor with telemetry system for monitoring of saliva glucose: a novel cavitas sensor. Biosens Bioelectron. (2016) 84:106–11. doi: 10.1016/j.bios.2015.12.014 26725934

[135] García-Carmona L Martín A Sempionatto JR Moreto JR González MC Wang J. Pacifier biosensor: toward noninvasive saliva biomarker monitoring. Anal Chem. (2019) 91:13883–91. doi: 10.1021/acs.analchem.9b03379, PMID: 31573188

[136] Arakawa T Tomoto K Nitta H Toma K Takeuchi S Sekita T. A wearable cellulose acetate-coated mouthguard biosensor for *in vivo* salivary glucose measurement. Anal Chem. (2020) 92:12201–7. doi: 10.1021/acs.analchem.0c01201, PMID: 32927955

[137] Garg SK Kipnes M Castorino K Bailey TS Akturk HK Welsh JB. Accuracy and safety of Dexcom G7 continuous glucose monitoring in adults with diabetes. Diabetes Technol Ther. (2022) 24:373–80. doi: 10.1089/dia.2022.0011, PMID: 35157505 PMC9208857

[138] Cobry EC Berget C Messer LH Forlenza GP. Review of the Omnipod^®^ 5 automated glucose control system powered by horizon^™^ for the treatment of type 1 diabetes. Ther Deliv. (2020) 11:507–19. doi: 10.4155/tde-2020-0055, PMID: 32723002 PMC8097502

[139] Soni A Wright N Agwu JC Timmis A Drew J Kershaw M. A practical approach to continuous glucose monitoring (rtCGM) and FreeStyle Libre systems (isCGM) in children and young people with type 1 diabetes. Diabetes Res Clin Pract. (2022) 184:109196. doi: 10.1016/j.diabres.2022.109196, PMID: 35033598

[140] Quirós C Alonso-Carril N Rodríguez-Rodríguez S Barahona M-J Orois A Simó-Servat A. The Medtronic 780G advanced hybrid closed-loop system achieves and maintains good glycaemic control in type 1 diabetes adults despite previous treatment. Endocrinol Diabetes Nutr. (2023) 70:130–5. doi: 10.1016/j.endien.2022.10.005, PMID: 36925230

[141] El-Khatib FH Balliro C Hillard MA Magyar KL Ekhlaspour L Sinha M. Home use of a bihormonal bionic pancreas versus insulin pump therapy in adults with type 1 diabetes: a multicentre randomised crossover trial. Lancet. (2017) 389:369–80. doi: 10.1016/S0140-6736(16)32567-3, PMID: 28007348 PMC5358809

[142] Castellanos LE Balliro CA Sherwood JS Jafri R Hillard MA Greaux E. Performance of the insulin-only iLet bionic pancreas and the bihormonal iLet using dasiglucagon in adults with type 1 diabetes in a home-use setting. Diabetes Care. (2021) 44:e118–20. doi: 10.2337/dc20-1086, PMID: 33906916 PMC8247518

[143] Park J Park N Han S Lee Y-B Kim G Jin S-M. A 4-week, two-center, open-label, single-arm study to evaluate the safety and efficacy of EOPatch in well-controlled type 1 diabetes mellitus. Diabetes Metab J. (2022) 46:941–7. doi: 10.4093/dmj.2021.0299, PMID: 35255546 PMC9723198

[144] Rebec M Cai K Dutt-Ballerstadt R Anderson E. A prospective multicenter clinical performance evaluation of the C-CGM system. J Diabetes Sci Technol. (2022) 16:390–6. doi: 10.1177/1932296820964574, PMID: 33084416 PMC8861779

[145] Baliga BS Tillman JB Olson B Vaughan S Sheikh FN Malone JK. First real-world experience with bigfoot Unity: a 6-month retrospective analysis. Clin Diabetes. (2023) 41:539–48. doi: 10.2337/cd22-0126 37849519 PMC10577513

[146] Schneider A Ypsomed A. How users distinguish between self-injection device platform variants. ONdrugDelivery. (2020) 2020:24–8.

[147] Sangave NA Aungst TD Patel DK. Smart connected insulin pens, caps, and attachments: a review of the future of diabetes technology. Diabetes Spectr. (2019) 32:378–84. doi: 10.2337/ds18-0069, PMID: 31798296 PMC6858073

[148] Sempionatto JR Moon J-M Wang J. Touch-based fingertip blood-free reliable glucose monitoring: personalized data processing for predicting blood glucose concentrations. ACS Sens. (2021) 6:1875–83. doi: 10.1021/acssensors.1c00139 33872007

[149] Bailey TS Alva S. Landscape of continuous glucose monitoring (CGM) and integrated CGM: accuracy considerations. Diabetes Technol Ther. (2021) 23:S-5–S-11. doi: 10.1089/dia.2021.0236 34546084

[150] Sachdeva P Shukla R Sahani A. A review on artificial pancreas and regenerative medicine used in the management of type 1 diabetes mellitus. J Med Eng Technol. (2022) 46:693–702. doi: 10.1080/03091902.2022.2095049, PMID: 35801984

[151] Russell SJ El-Khatib FH Sinha M Magyar KL McKeon K Goergen LG. Outpatient glycemic control with a bionic pancreas in type 1 diabetes. N Engl J Med. (2014) 371:313–25. doi: 10.1056/NEJMoa1314474, PMID: 24931572 PMC4183762

[152] Schliess F Heise T Benesch C Mianowska B Stegbauer C Broge B. Artificial pancreas systems for people with type 2 diabetes: conception and design of the European CLOSE project. J Diabetes Sci Technol. (2019) 13:261–7. doi: 10.1177/1932296818803588, PMID: 30241444 PMC6399797

[153] Taleb N Carpentier AC Messier V Ladouceur M Haidar A Rabasa-Lhoret R. Efficacy of artificial pancreas use in patients with type 2 diabetes using intensive insulin therapy: a randomized crossover pilot trial. Diabetes Care. (2019) 42:e107–9. doi: 10.2337/dc18-2406, PMID: 31221705

[154] Daly AB Boughton CK Nwokolo M Hartnell S Wilinska ME Cezar A. Fully automated closed-loop insulin delivery in adults with type 2 diabetes: an open-label, single-center, randomized crossover trial. Nat Med. (2023) 29:203–8. doi: 10.1038/s41591-022-02144-z, PMID: 36631592 PMC9873557

[155] Alves TMR Deroco PB Wachholz Junior D Vidotto LHB Kubota LT. Wireless wearable electrochemical sensors: a review. Braz J Anal Chem. (2021) 8. doi: 10.30744/brjac.2179-3425.RV-62-2020

[156] Fan R Andrew TL. Perspective—challenges in developing wearable electrochemical sensors for longitudinal health monitoring. J Electrochem Soc. (2020) 167:037542. doi: 10.1149/1945-7111/ab67b0

[157] Xu L Zhou Z Fan M Fang X. Advances in wearable flexible electrochemical sensors for sweat monitoring: a mini-review. Int J Electrochem Sci. (2023) 18:13–9. doi: 10.1016/j.ijoes.2023.01.009

[158] Teymourian H Parrilla M Sempionatto JR Montiel NF Barfidokht A Van Echelpoel R. Wearable electrochemical sensors for the monitoring and screening of drugs. ACS Sens. (2020) 5:2679–700. doi: 10.1021/acssensors.0c01318, PMID: 32822166

[159] Khosravi S Soltanian S Servati A Khademhosseini A Zhu Y Servati P. Screen-printed textile-based electrochemical biosensor for noninvasive monitoring of glucose in sweat. Biosensors. (2023) 13:684. doi: 10.3390/bios13070684, PMID: 37504083 PMC10377550

[160] Jeerapan I Poorahong S. Flexible and stretchable electrochemical sensing systems: materials, energy sources, and integrations. J Electrochem Soc. (2020) 167:037573. doi: 10.1149/1945-7111/ab7117

[161] Bandodkar AJ Jia W Wang J. Tattoo-based wearable electrochemical devices: a review. Electroanalysis. (2015) 27:562–72. doi: 10.1002/elan.201400537

[162] Jeerapan I Sempionatto JR Pavinatto A You J-M Wang J. Stretchable biofuel cells as wearable textile-based self-powered sensors. J Mater Chem A. (2016) 4:18342–53. doi: 10.1039/C6TA08358G, PMID: 28439415 PMC5400293

[163] Lee H Choi TK Lee YB Cho HR Ghaffari R Wang L. A graphene-based electrochemical device with thermoresponsive microneedles for diabetes monitoring and therapy. Nat Nanotechnol. (2016) 11:566–72. doi: 10.1038/nnano.2016.38, PMID: 26999482

[164] Ning Q Feng S Sun Q Yu R Li T Xu H. Finger-actuated wireless-charging wearable multifunctional sweat-sensing system for levodopa and vitamin C. Nano Res. (2023) 17:3096–106. doi: 10.1007/s12274-023-6197-6

[165] Bandodkar AJ Jia W Yardımcı C Wang X Ramirez J Wang J. Tattoo-based noninvasive glucose monitoring: a proof-of-concept study. Anal Chem. (2015) 87:394–8. doi: 10.1021/ac504300n, PMID: 25496376

[166] Kim J Sempionatto JR Imani S Hartel MC Barfidokht A Tang G. Simultaneous monitoring of sweat and interstitial fluid using a single wearable biosensor platform. Adv Sci. (2018) 5:1800880. doi: 10.1002/advs.201800880, PMID: 30356971 PMC6193173

[167] Yu Y Nassar J Xu C Min J Yang Y Dai A. Biofuel-powered soft electronic skin with multiplexed and wireless sensing for human-machine interfaces. Sci Robot. (2020) 5:eaaz7946. doi: 10.1126/scirobotics.aaz7946 32607455 PMC7326328

[168] Bandodkar AJ You J-M Kim N-H Gu Y Kumar R Mohan AV. Soft, stretchable, high power density electronic skin-based biofuel cells for scavenging energy from human sweat. Energy Environ Sci. (2017) 10:1581–9. doi: 10.1039/C7EE00865A

[169] Ciui B Martin A Mishra RK Brunetti B Nakagawa T Dawkins TJ. Wearable wireless tyrosinase bandage and microneedle sensors: toward melanoma screening. Adv Healthc Mater. (2018) 7:e1701264. doi: 10.1002/adhm.201701264, PMID: 29345430

[170] Martín A Kim J Kurniawan JF Sempionatto JR Moreto JR Tang G. Epidermal microfluidic electrochemical detection system: enhanced sweat sampling and metabolite detection. ACS Sens. (2017) 2:1860–8. doi: 10.1021/acssensors.7b00729, PMID: 29152973

[171] Bandodkar AJ Hung VW Jia W Valdés-Ramírez G Windmiller JR Martinez AG. Tattoo-based potentiometric ion-selective sensors for epidermal pH monitoring. Analyst. (2013) 138:123–8. doi: 10.1039/C2AN36422K, PMID: 23113321

[172] Jia W Bandodkar AJ Valdés-Ramírez G Windmiller JR Yang Z Ramírez J. Electrochemical tattoo biosensors for real-time noninvasive lactate monitoring in human perspiration. Anal Chem. (2013) 85:6553–60. doi: 10.1021/ac401573r, PMID: 23815621

[173] Sempionatto JR Nakagawa T Pavinatto A Mensah ST Imani S Mercier P. Eyeglasses based wireless electrolyte and metabolite sensor platform. Lab Chip. (2017) 17:1834–42. doi: 10.1039/C7LC00192D, PMID: 28470263 PMC5507201

[174] Wang M Yang Y Min J Song Y Tu J Mukasa D. A wearable electrochemical biosensor for the monitoring of metabolites and nutrients. Nat Biomed Eng. (2022) 6:1225–35. doi: 10.1038/s41551-022-00916-z, PMID: 35970928 PMC10432133

[175] Tehrani F Teymourian H Wuerstle B Kavner J Patel R Furmidge A. An integrated wearable microneedle array for the continuous monitoring of multiple biomarkers in interstitial fluid. Nat Biomed Eng. (2022) 6:1214–24. doi: 10.1038/s41551-022-00887-1, PMID: 35534575

[176] Valdés-Ramírez G Li Y-C Kim J Jia W Bandodkar AJ Nuñez-Flores R. Microneedle-based self-powered glucose sensor. Electrochem Commun. (2014) 47:58–62. doi: 10.1016/j.elecom.2014.07.014

[177] Lv J Jeerapan I Tehrani F Yin L Silva-Lopez CA Jang J-H. Sweat-based wearable energy harvesting-storage hybrid textile devices. Energy Environ Sci. (2018) 11:3431–42. doi: 10.1039/C8EE02792G

[178] Parrilla M Cánovas R Jeerapan I Andrade FJ Wang J. A textile-based stretchable multi-ion potentiometric sensor. Adv Healthc Mater. (2016) 5:996–1001. doi: 10.1002/adhm.201600092, PMID: 26959998 PMC4936408

[179] Yin L Kim KN Lv J Tehrani F Lin M Lin Z. A self-sustainable wearable multi-modular E-textile bioenergy microgrid system. Nat Commun. (2021) 12:1542. doi: 10.1038/s41467-021-21701-7, PMID: 33750816 PMC7943583

[180] Ye S Feng S Huang L Bian S. Recent progress in wearable biosensors: from healthcare monitoring to sports analytics. Biosensors. (2020) 10:205. doi: 10.3390/bios10120205, PMID: 33333888 PMC7765261

[181] Sempionatto JR Montiel VR-V Vargas E Teymourian H Wang J. Wearable and mobile sensors for personalized nutrition. ACS Sens. (2021) 6:1745–60. doi: 10.1021/acssensors.1c00553, PMID: 34008960

[182] Tang W Yin L Sempionatto JR Moon JM Teymourian H Wang J. Touch-based stressless cortisol sensing. Adv Mater. (2021) 33:e2008465. doi: 10.1002/adma.202008465, PMID: 33786887

[183] Moon JM Teymourian H De la Paz E Sempionatto JR Mahato K Sonsa-ard T. Non-invasive sweat-based tracking of L-dopa pharmacokinetic profiles following an oral tablet administration. Angew Chem Int Ed. (2021) 60:19074–8. doi: 10.1002/anie.202106674, PMID: 34145703 PMC8373796

[184] Moon J-M Del Cano R Moonla C Sakdaphetsiri K Saha T Lc FM. Self-testing of ketone bodies, along with glucose, using touch-based sweat analysis. ACS Sens. (2022) 7:3973–81. doi: 10.1021/acssensors.2c02369, PMID: 36512725

[185] Teymourian H Tehrani F Mahato K Wang J. Lab under the skin: microneedle based wearable devices. Adv Healthc Mater. (2021) 10:e2002255. doi: 10.1002/adhm.202002255, PMID: 33646612

[186] Teymourian H Moonla C Tehrani F Vargas E Aghavali R Barfidokht A. Microneedle-based detection of ketone bodies along with glucose and lactate: toward real-time continuous interstitial fluid monitoring of diabetic ketosis and ketoacidosis. Anal Chem. (2019) 92:2291–300. doi: 10.1021/acs.analchem.9b05109 31874029

[187] Goud KY Moonla C Mishra RK Yu C Narayan R Litvan I. Wearable electrochemical microneedle sensor for continuous monitoring of levodopa: toward Parkinson management. ACS Sens. (2019) 4:2196–204. doi: 10.1021/acssensors.9b01127, PMID: 31403773

[188] Shuvo MMH Titirsha T Amin N Islam SK. Energy harvesting in implantable and wearable medical devices for enduring precision healthcare. Energies. (2022) 15:7495. doi: 10.3390/en15207495

[189] Kumar R Johnson KM Williams NX Subramanian V. Scaling printable Zn–Ag_2_O batteries for integrated electronics. Adv Energy Mater. (2019) 9:1803645. doi: 10.1002/aenm.201803645

[190] Yin L Cao M Kim KN Lin M Moon J-M Sempionatto JR. A stretchable epidermal sweat sensing platform with an integrated printed battery and electrochromic display. Nat Electron. (2022) 5:694–705. doi: 10.1038/s41928-022-00843-6

[191] Salani M Roy S Fissell WH IV. Innovations in wearable and implantable artificial kidneys. Am J Kidney Dis. (2018) 72:745–51. doi: 10.1053/j.ajkd.2018.06.005 30146422

[192] Jena R Aggarwal A Choudhary GR Bajpai NK. Current status and future of artificial kidney in humans. Indian J Nephrol. (2022) 32:531. doi: 10.4103/ijn.ijn_240_21 36704585 PMC9872927

[193] Torrente-Rodríguez RM Lukas H Tu J Min J Yang Y Xu C. SARS-CoV-2 RapidPlex: a graphene-based multiplexed telemedicine platform for rapid and low-cost COVID-19 diagnosis and monitoring. Matter. (2020) 3:1981–98. doi: 10.1016/j.matt.2020.09.027, PMID: 33043291 PMC7535803

[194] Baskin DS Sharpe MA Nguyen L Helekar SA. Case report: End-stage recurrent glioblastoma treated with a new noninvasive non-contact oncomagnetic device. Front Oncol. (2021) 11:708017. doi: 10.3389/fonc.2021.708017 34367992 PMC8341943

[195] Dornhoffer JR Lawlor SK Saoji AA Driscoll CL. Initial experiences with the Envoy Acclaim^®^ fully implanted cochlear implant. J Clin Med. (2023) 12:5875. doi: 10.3390/jcm12185875, PMID: 37762817 PMC10532076

[196] Lee H Lee Y Song C Cho HR Ghaffari R Choi TK. An endoscope with integrated transparent bioelectronics and theranostic nanoparticles for colon cancer treatment. Nat Commun. (2015) 6:10059. doi: 10.1038/ncomms10059, PMID: 26616435 PMC4674684

[197] Bajer L Ryou M Thompson CC Drastich P. Novel upper gastrointestinal bleeding sensor capsule: a first human feasibility and safety trial. Gastroenterology. (2022) 57:203–8. doi: 10.5946/ce.2023.111 PMC10984735 38229441

[198] Reynolds J Ahmmed P Bozkurt A. An injectable system for subcutaneous photoplethysmography, accelerometry, and thermometry in animals. IEEE Trans Biomed Circuits Syst. (2019) 13:825–34. doi: 10.1109/TBCAS.2019.2923153, PMID: 31217129

[199] Bussooa A Neale S Mercer JR. Future of smart cardiovascular implants. Sensors. (2018) 18:2008. doi: 10.3390/s18072008 29932154 PMC6068883

[200] Tang J Wang J Huang K Ye Y Su T Qiao L. Cardiac cell-integrated microneedle patch for treating myocardial infarction. Sci Adv. (2018) 4:eaat9365. doi: 10.1126/sciadv.aat9365 30498778 PMC6261659

[201] Kwiatkowski A Borys S Sikorska K Drozdowska K Smulko JM. Clinical studies of detecting COVID-19 from exhaled breath with electronic nose. Sci Rep. (2022) 12:15990. doi: 10.1038/s41598-022-20534-8, PMID: 36163492 PMC9512806

